# Transcriptome profiling of a *Rhizobium leguminosarum* bv. *trifolii rosR* mutant reveals the role of the transcriptional regulator RosR in motility, synthesis of cell-surface components, and other cellular processes

**DOI:** 10.1186/s12864-015-2332-4

**Published:** 2015-12-29

**Authors:** Kamila Rachwał, Ewa Matczyńska, Monika Janczarek

**Affiliations:** Department of Genetics and Microbiology, Institute of Microbiology and Biotechnology, Maria Curie-Skłodowska University, Akademicka 19, 20-033 Lublin, Poland; Department of Mathematics and Computer Science, Institute of Computer Science, Jagiellonian University, Łojasiewicza 6, 30-348 Cracow, Poland; Genomed SA, Ponczowa 12, 02-971 Warsaw, Poland

**Keywords:** Transcriptional regulator RosR, *Rhizobium leguminosarum*, RNA sequencing, Transcriptome analysis, Motility, Cell-surface components, Polysaccharides, Nitrogen-fixing symbiosis

## Abstract

**Background:**

*Rhizobium leguminosarum* bv. *trifolii* is a soil bacterium capable of establishing a symbiotic relationship with red clover (*Trifolium pratense*). The presence of surface polysaccharides and other extracellular components as well as motility and competitiveness are essential traits for both adaptation of this bacterium to changing environmental conditions and successful infection of host plant roots. The *R. leguminosarum* bv. *trifolii rosR* gene encodes a protein belonging to the family of Ros/MucR transcriptional regulators, which contain a Cys_2_His_2_-type zinc-finger motif and are involved in the regulation of exopolysaccharide synthesis in several rhizobial species. Previously, it was established that a mutation in the *rosR* gene significantly decreased exopolysaccharide synthesis, increased bacterial sensitivity to some stress factors, and negatively affected infection of clover roots.

**Results:**

RNA-Seq analysis performed for the *R. leguminosarum* bv. *trifolii* wild-type strain Rt24.2 and its derivative Rt2472 carrying a *rosR* mutation identified a large number of genes which were differentially expressed in these two backgrounds. A considerable majority of these genes were up-regulated in the mutant (63.22 %), indicating that RosR functions mainly as a repressor. Transcriptome profiling of the *rosR* mutant revealed a role of this regulator in several cellular processes, including the synthesis of cell-surface components and polysaccharides, motility, and bacterial metabolism. Moreover, it was established that the Rt2472 strain was characterized by a longer generation time and showed an increased aggregation ability, but was impaired in motility as a result of considerably reduced flagellation of its cells.

**Conclusions:**

The comparative transcriptome analysis of *R. leguminosarum* bv. *trifolii* wild-type Rt24.2 and the Rt2472 mutant identified a set of genes belonging to the RosR regulon and confirmed the important role of RosR in the regulatory network. The data obtained in this study indicate that this protein affects several cellular processes and plays an important role in bacterial adaptation to environmental conditions.

**Electronic supplementary material:**

The online version of this article (doi:10.1186/s12864-015-2332-4) contains supplementary material, which is available to authorized users.

## Background

Rhizobia are a unique group of bacteria which can either exist as free-living organisms in soil or establish a nitrogen-fixing symbiosis with legumes [1, 2]. This type of plant–microbe interaction is essential for the functioning of the biosphere, since it provides nutrients to plants and increases soil fertility and field crops. However, before rhizobia can find a compatible host plant, they often must survive long periods of time as free-living bacteria in the soil. In such periods, rhizobial cells are exposed to the action of environmental factors such as nutrient limitation, pH, salinity, drought, temperature variation, heavy metals, and oxidative stress [3–9]. In order to adapt to these conditions, rhizobia have developed a wide range of strategies and features that allow them to survive in the soil. One of the most important of these adaptations is the composition of the bacterial envelope. The outer surface of these bacteria contains various polysaccharides (PSs), such as lipopolysaccharide (LPS), capsular polysaccharide (CPS) and cellulose fibrils, as well as the neutral polysaccharide (NP, glucomannan) and gel-forming polysaccharide (GPS) recently reported in *Rhizobium leguminosarum* [10–12]. NP, cellulose fibrils, and LPS are required for attachment to and biofilm formation on soil particles and plant roots, as well as successful infection of the host plant and adaptation to the conditions prevailing inside the nodule, a specific organ formed on legume roots [13–17].

In addition, exopolysaccharide (EPS), secreted in large amounts by rhizobia and weakly associated with their cells, plays an important protective role against desiccation and other stress conditions occurring in the soil. This polymer is also crucial for the attachment of rhizobia to abiotic as well as biotic surfaces, including plant roots, and establishment of an effective symbiosis [11, 12, 18, 19]. Another type of PS synthesized by these bacteria is β-glucan, which is found in their periplasmic space and is required for adaptation to hypo-osmotic conditions [20–22].

Apart from the influence of envelope components, bacterial adaptation to changing soil conditions and interaction with plants are affected by some of the proteins that rhizobia secrete into the environment, including those exported via Type I secretion system (e.g. NodO, glycanases, adhesins, and substrate-binding proteins) [11, 23–25]. Some of these secreted proteins are required for the attachment of rhizobia to host plant roots, which is a very important step in the initiation of symbiosis. An *R. leguminosarum* protein called rhicadhesin plays a significant role in bacterial attachment to root hairs, particularly at alkaline pH [10, 11]. Other proteins involved in this process include PlyB glycanase (which affects EPS processing), cadherin-like proteins (calcium-binding adherence proteins), and *Rhizobium*-adhering proteins (Raps) secreted via Type I secretion system encoded in *R. leguminosarum* by the *prsD* and *prsE* genes [11, 23, 24]. Moreover, some proteins secreted by rhizobia into the environment allow the bacteria to survive in the absence of their legume hosts. Among these, several proteins associated with the uptake of various nutrients have been identified (e.g. proteins binding sorbitol, ribose and other sugars, leucine/isoleucine/valine, arginine, and dipeptides) [24].

Furthermore, motility of rhizobial cells is an essential trait for their survival in the environment as well as competitiveness [26–31]. In the soil, the availability of carbon sources utilizable by bacteria is limited, since most components of organic matter form complexes with other compounds, thus becoming resistant to bacterial degradation [32]. Because of this, the rhizosphere, a narrow soil region rich in various types of substances secreted by plant roots, is a very attractive niche for bacteria [11, 33, 34]. Therefore, the ability to sense nutrients and move towards them provides a competitive advantage for motile and chemotactic rhizobia over non-motile and non-chemotactic strains [28]. Several studies have shown that non-motile or non-chemotactic bacteria are less adaptive to changing environmental conditions and less competitive when it comes to plant root infection [27, 28, 35–38]. Additionally, swarming of rhizobial cells is affected by exudates from legume seeds [39].

*Rhizobium leguminosarum* bv*. trifolii* is a microsymbiont of red clover (*Trifolium pratense*). Previously, *rosR* encoding a regulatory protein which positively influenced EPS production was identified and characterized in this bacterium [40, 41]. A mutation in this gene resulted in a significant decrease in EPS synthesis [42]. *R. leguminosarum* bv*. trifolii* RosR belongs to the family of Ros/MucR transcriptional regulators, which are involved in the regulation of EPS synthesis in several rhizobial species, including *Sinorhizobium meliloti*, *Rhizobium etli*, and *Agrobacterium tumefaciens* [43–46]. RosR is a small protein (15.7 kDa) characterized by a Cys_2_His_2_-type zinc-finger motif, which is responsible for binding to a 22-bp-long sequence called the RosR-box. Previously, it was established that RosR recognized the RosR-box motif located in the *rosR* upstream region and negatively regulated the transcription of its own gene [40]. A *R. leguminosarum* bv. *trifolii* strain with a *rosR* mutation formed dry, wrinkled colonies on agar plates, which were significantly different from those formed by the wild-type bacteria. Furthermore, this mutant strain showed some changes in the O-chain of LPS. The *rosR* mutation also resulted in a decreased biofilm formation, a higher sensitivity to osmotic and oxidative stresses, and a significantly impaired symbiosis with clover plants (in comparison to the wild-type strain, the nodules formed were less numerous and were unable to fix nitrogen) [8, 47]. All these data suggest that RosR is an essential protein involved in several cellular processes and, possibly, an important element of the rhizobial regulatory network. Although previous results concerning the *rosR* mutant indicate that RosR is engaged in adaptation to stress conditions, it is still unknown how many genes are under the control of this transcriptional regulator.

In this study, we performed a comparative transcriptomic analysis of *R. leguminosarum* bv. *trifolii* wild-type 24.2 and its derivative mutant strain Rt2472*rosR*, which provided novel data on RosR-mediated regulation of gene expression in this bacterium. It was found that RosR influenced the expression of a large number of genes, including those related to the synthesis of cell-surface components and polysaccharides, motility, and metabolic pathways. Among these genes, a significant majority were up-regulated in the *rosR* mutant, suggesting that RosR functions in *R. leguminosarum* bv. *trifolii* cells mainly as a negative regulatory protein.

## Results and discussion

### RNA-Seq analysis of the wild-type strain Rt24.2 and its derivative *rosR* mutant Rt2472

Our previous studies of *R. leguminosarum* bv. *trifolii rosR* had suggested that this gene could play a global regulatory role in the functioning of rhizobial cells. As a further step in the investigation of this problem, in the present study, we used comparative transcriptomic analysis to establish the set of genes belonging to the RosR regulon. We compared wild-type strain Rt24.2 with its derivative, the Rt2472*rosR* mutant, obtained via random mutagenesis, which has a mini-Tn*5* transposon located inside the *rosR* coding region, between 151 bp and 152 bp of the open reading frame [42]. First, in order to identify the genetic bases of Rt24.2 and to use it as a reference strain to map the sequence reads from the transcriptome data sets, a draft genomic sequence of this strain was obtained (181 contigs with a total length of 7,653,217 bp, GC content = 60.6 %). The sequence of Rt24.2 was compared with genomes of closely related rhizobial strains (including *R. leguminosarum* bv*. viciae* 3841, *R. leguminosarum* bvs*. trifolii* WSM2304 and WSM1325, and *Rhizobium etli* CFN42). In total, 7,374 potential coding sequences were identified in the Rt24.2 genome. Among these, 7,149 sequences (96.95 %) were matched to *Rhizobium*, 141 (1.91 %) to other bacterial species, and only 84 (1.13 %) were strain-specific and were not matched to any rhizobia.

In order to compare the expression of these genes in the *rosR* mutant to their expression in the wild-type bacterium, three RNA-Seq libraries were prepared for the two strains grown in the same conditions. Qualitative analyses of RNA samples after depletion of 23S and 16S rRNA indicated that they were of high quality and that a great majority of rRNA had been removed. The mRNAs of the Rt24.2 and Rt2472 strains were sequenced using Illumina MiSeq with SBS technology (general features of the individual runs are shown in Additional file 1A). On average, 5,368,924 reads (SD = 1,005,388) for the wild-type strain and 5,365,247 reads (SD = 1,864,858) for the *rosR* mutant were unambiguously mapped. This indicated that similar amounts of data were mapped for each strain. The results were subsequently analyzed using the Cuffdiff tool from the Cufflinks package to normalize the data and test for differential expression between the strains tested (Additional file 1B). Box plots generated in order to compare the distributions of RNA-Seq data between biological repetitions of an individual strain and between Rt24.2 and Rt2472 showed similar values for these strains (comparable distributions seen as similar box sizes and box plot whiskers) (Additional file 1C).

### Identification of genes differentially expressed in the *rosR* mutant Rt2472 and the wild-type strain Rt24.2

**Fig. 1:**
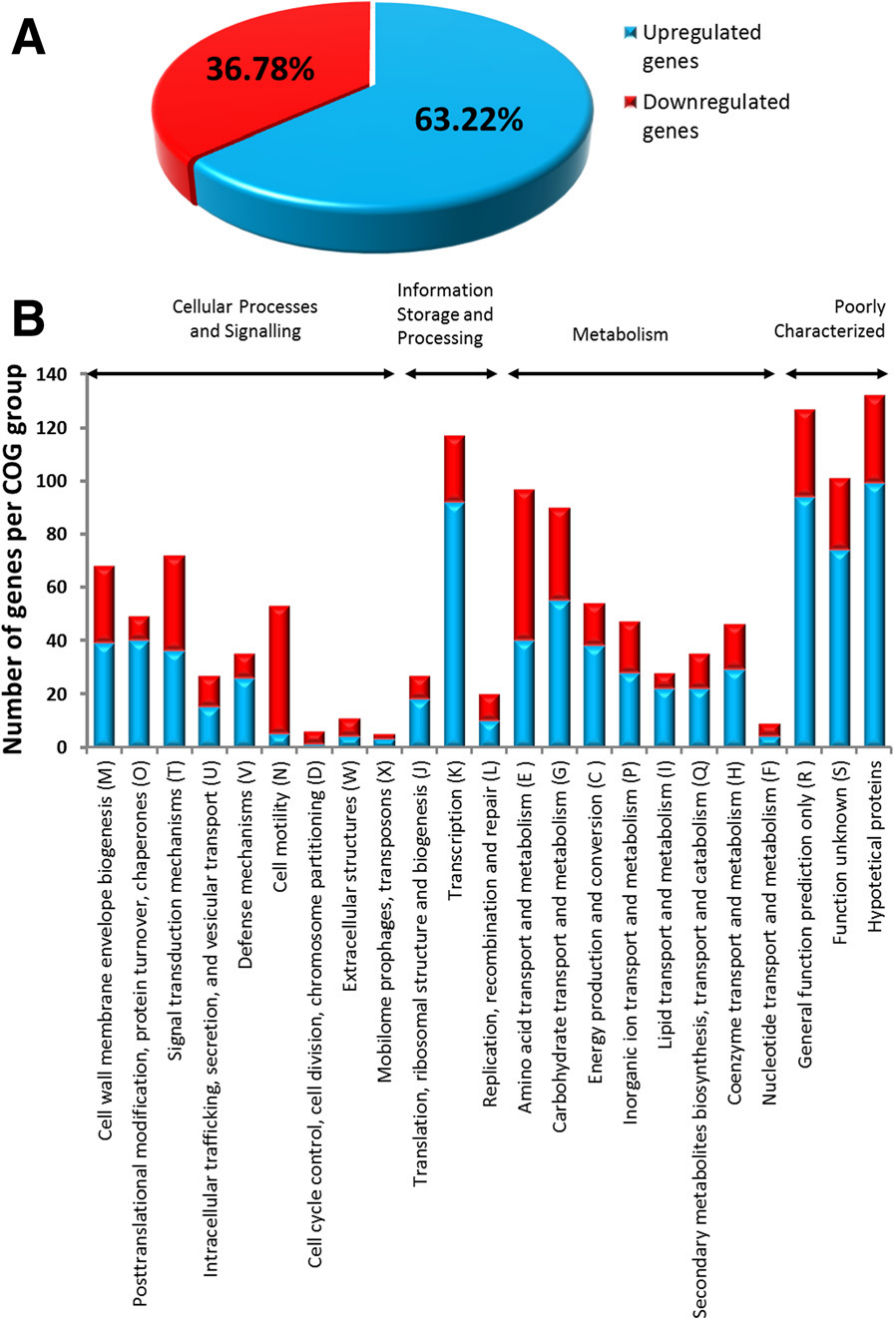
Genes differentially transcribed in the *rosR* mutant Rt2472 in relation to the wild-type *R. leguminosarum* bv*. trifolii* strain Rt24.2. **a** Global classification of the genes into up-regulated ones, whose expression was higher, and down-regulated ones, whose expression was lower in the *rosR* mutant than in the wild-type background. **b** Classification of the up-regulated and down-regulated genes in the *rosR* mutant into individual functional groups (COGs M-S)

An analysis of the functional composition of the Rt24.2 transcriptome showed that the most numerously represented categories were related to metabolism processes, especially the functional groups (COGs) G, E, P, and C, which are associated with the transport and metabolism of carbohydrates, amino acids, and inorganic ions as well as energy production and conversion, respectively (Fig. 1). Also, COG classes K (transcription), J (translation/ribosome structure and biogenesis), M (cell wall/membrane/envelope biogenesis), and the poorly characterized class R were highly represented in the *R. leguminosarum* transcriptome.

In order to establish which genes belonged to the RosR regulon in *R. leguminosarum*, transcriptional profiles for Rt24.2 and Rt2472 were obtained using the genome-wide RNA-Seq approach. The high reproducibility between the analyzed strains (evidenced by the high correlation factors of read counts) and the relative expression values of the RNA-Seq data allowed us to obtain *p-values* adjusted with Cuffdiff (Cufflinks package). A histogram of FPKM values (Fragments Per Kilobase of transcript per Million fragments mapped) for the genes, with three biological repetitions for each strain analyzed, is presented in Additional file 2A. A point diagram of FPKM values between the samples and a diagram indicating the dependence of *p-values* on fold changes are shown in Additional file 2B and C, respectively. Based on the fold changes of gene expressions obtained in the *rosR* mutant and the wild-type strain (log_2_ of mut/wt values >2), it was established that 1106 genes were transcribed at significantly different levels in these genetic backgrounds. This indicated that RosR influences the expression of many loci and plays an essential role in the rhizobial regulatory network (Additional file 3). Among the genes in question, a significant majority were up-regulated in the *rosR* mutant (63.22 %), whereas 36.78 % were down-regulated (Fig. 1a), suggesting that RosR functions mainly as a negative regulator of gene transcription in this bacterium. We successfully classified 89.48 % of the genes into particular COGs (Fig. 1b) [48]. Most of these genes belonged to the following groups: transcription (COG K) (9.33 %), general functions (R) (10.13 %), unknown function (S) (8.05 %), transport and metabolism of amino acids (E) (7.74 %) and carbohydrates (G) (7.18 %), signal transduction mechanisms (T) (5.74 %), cell wall/membrane/envelope biogenesis (M) (5.41 %), and cell motility (N) (4.23 %). Moreover, it was found that for a majority of the COGs (K, M, O, G, C, T, V, P, I, H, R, S and that with hypothetical functions) a significantly larger number of the genes were up-regulated than down-regulated in the *rosR* mutant in relation to the parental strain (Fig. 1b). In contrast, a great majority of the genes belonging to COGs N and E were expressed at significantly lower levels in the *rosR* mutant versus the wild-type, indicating that RosR positively affected the transcription of these genes.

**Fig. 2:**
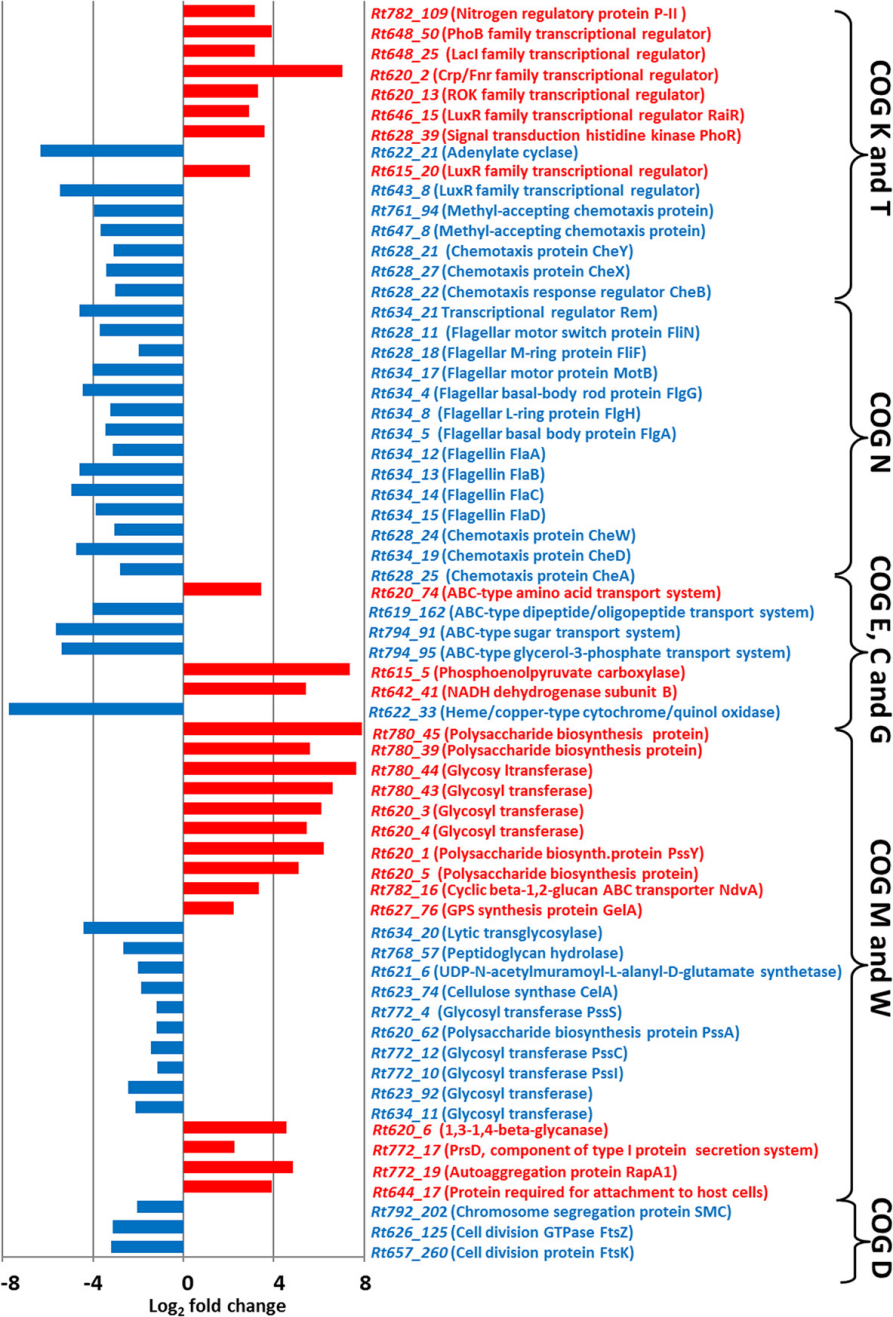
Representative genes of *R. leguminosarum* bv*. trifolii* from the individual COG groups differentially expressed in the Rt2472 mutant in relation to the wild-type strain Rt24.2. Descriptions of putative functions of the protein products encoded by these genes are given in brackets

**Fig. 3:**
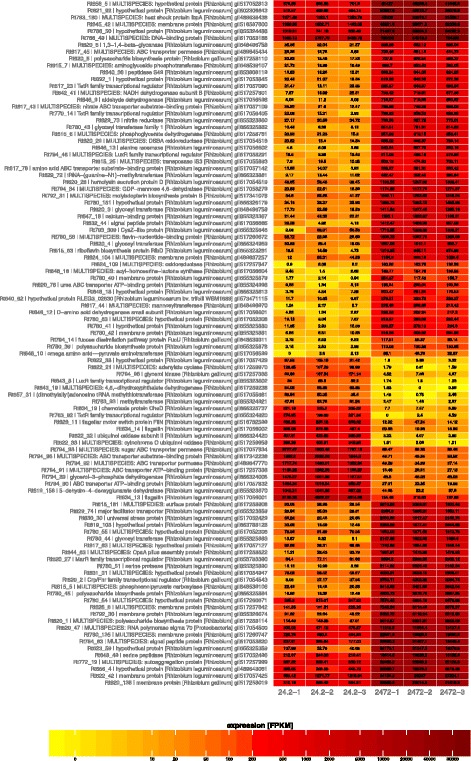
Heatmap of FPKM values showing differences in expression between the *rosR* mutant Rt2472 and the wild-type strain Rt24.2 for 100 genes with the highest log_2_ fold change values. The biological repetitions for Rt24.2 are marked with Rt24.2-1, Rt24.2-2 and Rt24.2-3, whereas those for Rt2472 are marked with Rt2472-1, Rt2472-2 and Rt2472-3

When the individual genes belonging to the RosR regulon were analyzed, the highest fold changes in mut/wt expression were found for *Rt780_45* encoding a periplasmic protein involved in polysaccharide export (log_2_ mut/wt = 7.89), *Rt622_32* and *Rt622_33* encoding subunits of a cytochrome/quinol oxidase (−6.94 and −7.72, respectively), *Rt780_44* encoding a glycosyl transferase involved in cell wall biogenesis (7.64), *Rt620_2* encoding a transcriptional regulator from the Crp/Fnr family (7.05), and *Rt622_21* encoding an adenylate cyclase (-6.32) (Fig. 2). A heatmap for 100 genes with the highest fold change values is shown in Fig. 3. The characteristics and putative functions of these genes are specified in Additional file 4.

### Transcription factors and signal transduction mechanisms

A functional category that was highly represented in *R. leguminosarum* bv. *trifolii* and differentially expressed in the Rt2472 mutant and the parental strain Rt24.2 was transcription (COG K) (Fig. 1b). A significant majority of the genes from this group were up-regulated (92), with only 25 genes being down-regulated in the *rosR* mutant. These genes encoded transcriptional factors belonging to various families such as Crp/Fnr, LysR, TetR, LysR, AraC, LuxR, MarR, and GntR. Among these proteins, Rt782_109 (P-II), Rt648_50 (PhoB), Rt648_25 (LacI), Rt620_2 (Crp), Rt620_13 (ROK), and Rt646_15 (RaiR) seem to be the most interesting (Fig. 2). The response regulator PhoB (Rt648_50) together with the sensor kinase PhoR (Rt628_39) form a two-component regulatory system used by rhizobial cells to recognize and adapt to phosphate starvation [49]. The catabolic protein Crp, the ROK protein binding Crp and cAMP, the LacI protein and the adenylate cyclase Rt622_21 are most probably engaged in the regulation of carbon metabolism. Rt782_109 (P-II) is highly homologous to GlnK proteins involved in nitrogen regulation in *R. etli* and *S. meliloti* [50], whereas Rt646_15 is identical to the LuxR-type regulator RaiR of *R. leguminosarum* bv. *viciae* engaged in quorum sensing [51]. A transcriptome analysis performed previously for an *S. meliloti glnK* mutant indicated that GlnK, which belongs to P-II proteins, is an important component of the regulatory cascade involved in nitrogen stress adaptation in rhizobial cells [50]. Also, several genes encoding LuxR-type regulators (e.g. Rt615_20 and Rt643_8), which possess a CheY-like domain responsible for binding chemotaxis proteins were found in this group (Fig. 2).

Moreover, many genes from COG T, involved in signal transduction mechanisms, were differentially expressed in the two genetic backgrounds studied, and a majority of them were down-regulated in the *rosR* mutant (Figs. 1-2, Additional file 4). Among them, there were several genes coding for methyl-accepting chemotaxis proteins (e.g. Rt761_94 and Rt647_8), chemotaxis proteins Rt628_21 (CheY) and Rt628_27 (CheX), and the response regulator Rt628_22 (CheB), which seem to play a significant role in signaling. All these data indicate that RosR affects the expression of many genes related to the response of rhizobial cells to environmental conditions (nitrogen, phosphate and carbon starvation) and quorum sensing.

### Cell motility and chemotaxis

Among the functional groups analyzed in this study, COG N is unique because of the fact that a great majority of the genes (90.6 %) associated with cell motility and chemotaxis were down-regulated in the *rosR* mutant compared to the wild-type strain (Fig. 1b). This indicated that RosR positively affected this trait in *R. leguminosarum*. Among these genes were those encoding the transcriptional regulator Rt634_21 (Rem), flagellar biosynthesis proteins (e.g. Rt628_11 (FliN) and Rt628_18 (FliF)), the flagellar motor protein Rt634_17 (MotB), as well as other flagellar proteins (Rt634_4 (FlgG), Rt634_8 (FlgH), and Rt634_5 (FlgA)). Moreover, transcription of *Rt634_12* (*flaA*)*, Rt634_13* (*flaB*)*, Rt634_14* (*flaC*)*,* and *Rt634_15* (*flaD*) genes encoding flagellins A, B, C, and D, respectively, was significantly decreased in the *rosR* mutant (Fig. 2).

In addition, several genes involved in chemotaxis were down-regulated in the *rosR* mutant. Apart from the MCP-type chemoreceptor genes mentioned earlier, also *Rt628_24*, *Rt634_19,* and *Rt628_25* genes, showing a high homology to *cheW, cheD,* and *cheA,* respectively, of *R. leguminosarum* bv. *viciae* strains 3841 and VF39 were found in this group (Fig. 2) [27–29].

Previously, Tambalo and others [28, 29] had identified two chemotaxis gene clusters (*che1* and *che2*) involved in the motility of *R. leguminosarum* and found that the *che1* cluster was the major genetic region that controlled swimming and chemotaxis (the *che2* cluster had a minor effect on chemotaxis). The first cluster contained such chemotaxis genes as *mcpC, cheY, cheA, cheW, cheR, cheB,* and *cheD.* Moreover, *flaA, flaB, flaC, flaD, motA, motB, motC*, *fli,* and *flg* genes, as well as the regulatory genes *rem, visR,* and *visN* were located in this region. Protein products of *flaA, flaB*, and *flaC* proved to be major components of the flagellar filament [28]. As reported earlier, LuxR-type regulators VisN/VisR and the Rem protein are components of the regulatory cascade involved in the expression of many flagellar, motility, and chemotaxis genes in *R. leguminosarum*. A great majority of these genes (*flaA, flaB, flaC, flaD, motA, motB, mcpD,* and the *che1* operon) are under positive regulation by this cascade [28, 29]. In this study, we found that the expression of many of these motility and chemotaxis genes was also positively regulated by RosR, which suggests that this protein may be an important element of this regulatory cascade.

### Carbon and nitrogen transport and metabolism, and energy production

In the *rosR* mutant, the expression of a large group of genes related to bacterial metabolism was significantly changed as well (Fig. 1b). This was especially visible in the case of the genes classified to COGs E (97 genes), G (90 genes), and C (54 genes). Many of these genes encoded enzymes and components of various transport systems. In group E, changes in expression were particularly evident in the case of genes encoding two transport systems (Fig. 2, Additional file 4): the expression of genes *Rt620_74, Rt620_75, Rt620_76,* and *Rt620_78* coding for components of the ABC-type branched-chain amino acid transport system was strongly up-regulated, whereas transcription of genes *Rt619_162, Rt619_ 163, Rt619_164,* and *Rt619_165* encoding components of the ABC-type dipeptide/oligopeptide transport system was down-regulated in the *rosR* mutant.

Similarly in the case of group G, expression of many genes for sugar transport systems was significantly decreased in the mutant background. Among them, the most important role seems to be played by *Rt794_91* and *Rt794_95* (Fig. 2).

In addition, large differences in expression levels between the mutant and the wild-type cells were found for genes encoding different enzymes (e.g. *Rt615_5* coding for a putative phosphoenolpyruvate carboxylase, *Rt642_41* coding for a NADH dehydrogenase, and *Rt622_33* coding for a cytochrome/quinol oxidase) (Fig. 2).

The differences observed in the expression of group G, E, and C genes suggested large disturbances in the cellular metabolism of the *rosR* mutant. These results are in agreement with the data obtained previously in Biolog tests which indicated that the *rosR* mutant utilized fewer carbon and nitrogen sources, and some of them utilized less effectively than the parental strain [47].

Several authors have reported that the ability to utilize numerous carbon and energy sources plays a very important role in both adaptation of rhizobia to soil conditions and their competitiveness in host plant infection. For example, catabolism of homoserine, a substantial component of pea root exudate, was linked with competiveness for nodulation of this host plant, as evidenced for *R. leguminosarum* bv. *viciae* 3841 [52]. On the other hand, an inability to catabolize galactose led to an increased ability to compete for nodule occupancy in *S. meliloti* [53].

### Cell-surface component synthesis and cell envelope biogenesis

Several genes associated with cell envelope biogenesis and synthesis of various polysaccharides and other surface components were differentially expressed in the analyzed strains (Fig. 1b). In this functional group (COG M), high up-regulation in the *rosR* mutant was established for the following loci: *Rt780_45* and *Rt780_39* encoding polysaccharide biosynthesis proteins, and *Rt780_44* and *Rt780_43* encoding glycosyl transferases (Fig. 2). These genes are located in the same genetic region and are probably engaged in LPS biosynthesis. Similarly, expression of some genes possibly involved in LPS or cell-wall biosynthesis, which are clustered in another genomic region called Pss-II, was highly increased in the mutant background. Among these were *Rt620_3* and *Rt620_4* coding for glycosyl transferases, and *Rt620_1* and *Rt620_5* coding for polysaccharide biosynthesis proteins, homologous to *pssY* and *pssL2* of the TA1 strain, respectively [54].

In the wild-type Rt24.2, RosR also decreased the expression of *Rt782_16*, encoding the cyclic β-1,2-glucan ABC transporter NdvA, and *Rt627_76,* which shows a high homology to the *RL4404 (gelA)* gene involved in GPS synthesis in *R. leguminosarum* bv. *viciae* 3841 (Fig. 2). β-1,2-glucan is required for adaptation of rhizobia to hypo-osmotic conditions, motility, and efficient symbiosis with host plants, whereas GPS is important for attachment to root hairs and competitive nodule infection [14, 21, 22].

In contrast, several genes potentially involved in peptidoglycan biosynthesis (*Rt634_20*, *Rt768_57* and *Rt621_6*) and formation of cellulose fibrils (*Rt623_74*), as well as those encoding other glycosyl transferases of unknown functions (e.g. *Rt623_92* and *Rt634_11*) were down-regulated in the *rosR* mutant (Fig. 2) [14].

Previous studies concerning RosR had indicated that this protein was a positive regulator of EPS synthesis in *R. leguminosarum* [40]. Therefore, we had expected that expression of many of the genes participating in this process, among them a significant majority of those located in the chromosomal cluster named Pss-I [54], would be positively affected by this regulator. Surprisingly, it was found that only a few of these genes encoding glycosyl transferases (*Rt772_4* (*pssS*), *Rt772_10* (*pssI*), *Rt772_12* (*pssC*), and *Rt620_62* (*pssA*)), were down-regulated in the mutant background, and the level of this regulation was very low (log_2_ fold change from −1.45 to −1.12) (Fig. 2). This finding suggests that the observed levels of regulation of *pss* gene transcription by RosR are sufficient for proper synthesis of EPS in *R. leguminosarum.* However, it cannot be excluded that RosR exerts a positive influence on the expression of other genes linked with EPS synthesis, whose function in this process has not yet been established. Previously, we had found that the expression of *pssA*, which encodes a glycosyl transferase involved in the first step of EPS synthesis, was positively regulated by RosR, but the observed effect was not high (a ~2-fold difference in the expression between the *rosR* mutant and the wild-type strain) [40]. A similar observation was made for MucR, a positive regulator of succinoglycan synthesis in *S. meliloti* which shows a significant similarity to the *R. leguminosarum* RosR; it was established that this protein had an only slight effect on the expression of the genes involved in this process (namely, it increased the transcription of *exoYFQ* and *exoK* and decreased the expression of *exoH* and *exoX* genes) [44].

In summary, the data obtained in this study indicate that RosR regulates the expression of many genes related to the synthesis of various rhizobial polysaccharides. In the wild-type cells, this protein represses the transcription of several genes involved in the biosynthesis of LPS, β-glucan, GPS and/or CPS, while positively regulates the expression of genes which participate in peptidoglycan, cellulose and EPS synthesis. All these components play an essential role in the proper functioning of the bacterial envelope and adaptation of the bacteria to environmental conditions [11, 54].

Furthermore, RosR proved to be a negative regulator of the expression of several genes from COG M, which encode proteins engaged in the processing of extracellular polysaccharides and in bacterial behavior. Among them, the most interesting genes were *Rt620_6* encoding a 1,3-1,4-β-glycanase, *Rt772_17* encoding component PrsD of the Type I protein secretion system, *Rt772_19* coding for an autoaggregation protein, and *Rt644_17* coding for a protein required for bacterial attachment to host plants (Fig. 2). The protein product of the *Rt772_19* gene exhibited a high homology to RapA1 agglutinin of *R. leguminosarum* bvs. *trifolii* and *viciae*, which belongs to the family of *Rhizobium*-adhering proteins (Raps) [24, 55].

The comparative RNA-Seq analysis of the *ros*R mutant and the wild-type strain revealed the role of the RosR protein in several cellular processes. Similar results had been obtained for *S. meliloti* strain 1021 with a mutation in *fadD* encoding a long-chain fatty acyl-coenzyme A ligase. In this latter strain, more than a thousand genes were identified as differentially expressed, including those for some metabolic activities, chemotaxis, motility, and iron uptake as well as stress-related genes [30]. Also, an RNA-Seq analysis of the *traI* and *ngrI* mutants of *S. fredii* NGR234 had identified a large set of genes which were differentially expressed in comparison to the parental strain (316 and 466 genes, respectively) [56]. These genes included, among others, those related to quorum sensing, motility and EPS biosynthesis. These data indicate that mutations in some essential genes in rhizobia may affect the expression of a huge number of other genes involved in various cellular processes.

### Generation time

It was established in this study that the mutation in *rosR* did not affect the genome structure or RNA processing (COGs A and B). However, some genes involved in cell division (COG D) were differentially expressed in the analyzed backgrounds (Fig. 1b). A majority of these genes were down-regulated in the *rosR* mutant (e.g. *Rt792_202* coding for a chromosome segregation protein and *Rt626_125* and *Rt657_260* genes coding for cell division proteins FtsZ and FtsK, respectively) (Fig. 2).

### Properties of *rosR* mutant cells

**Fig. 4:**
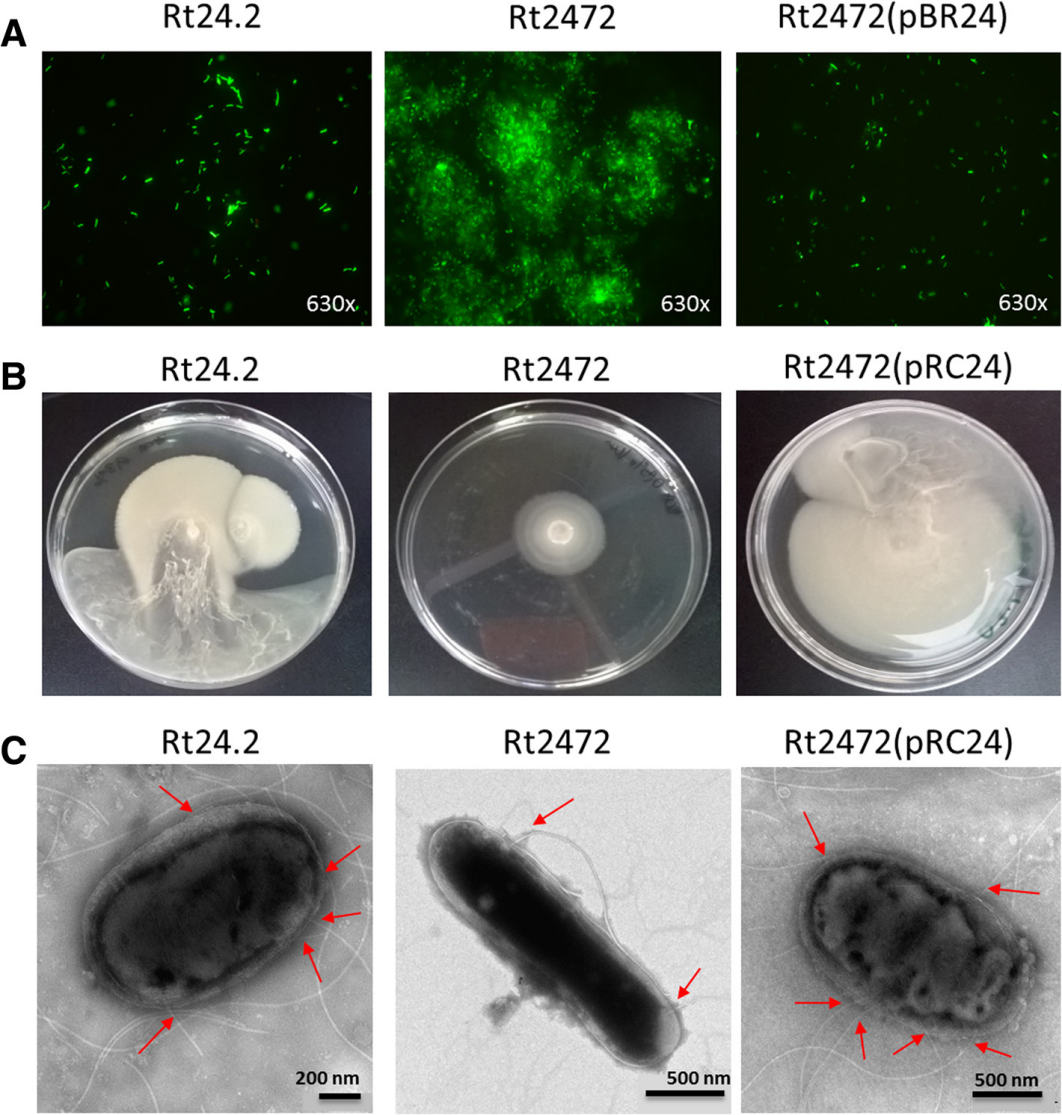
Properties of the *rosR* mutant Rt2472 in comparison to the wild-type Rt24.2 and Rt2472(pRC24) strains. **a** Morphology of bacterial cells tagged with the pHC60 plasmid containing a *gusA* reporter gene in fluorescence microscopy (magnification, 630×); **b** Motility of the analyzed strains determined on 0.3 % 79CA agar plates in a 3-week experiment; **c** The cells of Rt24.2, Rt2472 and Rt2472(pRC24) visualized in transmission electron microscopy (flagella are marked with *red arrows*)

In order to verify the data obtained from the RNA-Seq analysis, we characterized several traits of the *rosR* mutant. To do so, in addition to the wild-type Rt24.2 and the Rt2472 mutant strains, Rt2472 after complementation of the mutation by *rosR* introduced on the pRC24 plasmid was used. At first, it was established that the Rt2472 mutant grew slower than the Rt24.2 and Rt2472(pRC24) strains on solid as well as in liquid media. Therefore, the doubling time of these strains was determined. In this experiment, it was established that the *rosR* mutant had a longer generation time (6.0 ± 0.5 h) than the wild-type (4.5 ± 0.5 h) and the Rt2472(pRC24) (5.0 ± 0.5 h) cells. Moreover, Rt2472 growth on agar plates was visible as rough colonies forming clumps, which significantly differed from the mucoid colonies formed by both Rt24.2 and Rt2472(pRC24). Similarly, in the liquid cultures, Rt2472 showed a tendency to aggregate. It was found that the mutant cells aggregated more effectively than the wild-type cells (Rt2472 = 36.69 % ± 2.91, Rt24.2 = 9.74 % ± 0.78, Rt2472(pRC24) = 10.10 % ± 0.85). A more detailed structure of these clumps was established in fluorescent microscopy using *gfp*-tagged strains (Fig. 4a, Additional file 5). In cultures of Rt24.2 and Rt2472 complementant, single and non-aggregated cells were visible. In contrast, the strain with the *rosR* mutation formed large aggregates with many grouped cells, which considerably outnumbered the single cells present in the culture. Moreover, large amounts of extracellular matrix surrounding the cells inside the clumps were observed. This phenotype could be explained by the increased expression of genes coding for the agglutinin RapA1, PrsD of the Type I secretion system, as well as glycosyl transferases involved in the synthesis of surface polysaccharides in the mutant cells. Previous studies had reported that RapA1 is a calcium-binding, cell-surface-associated agglutinin, whose function is to adhere to the root hairs [56]. Moreover, a positive correlation between bacterial aggregation and biofilm formation had been observed, in which polysaccharides play a significant role [19, 57].

Apart from the differences described above, the *rosR* mutant showed significantly slower migration on both semi-solid and solid media in relation to both Rt24.2 and Rt2472(pRC24) (Fig. 4b, Additional file 6). This difference was especially visible on 0.3 % agar plates after 21 days of incubation. In order to establish whether the motility of this mutant was altered by flagella (more precisely, their number and/or structure), cells of the Rt2472, Rt24.2 and Rt2472(pRC24) strains were observed in transmission electron microscopy. The wild-type cells were rod-shaped, with an average length of 1.716 ± 0.38 μm (*n* = 15) (Rt2472(pRC24) = 1.792 ± 0.41 μm), and were peritrichously flagellated with 2–5 flagella (Fig. 4c). Moreover, these cells were surrounded by a thick and light layer of extracellular material, which was probably composed of EPS (Additional file 7). In contrast, the Rt2472 cells were slightly shorter (1.56 ± 0.15 μm). Furthermore, these cells were surrounded by a thinner but more densely packed extracellular layer than in the wild-type. In addition, *rosR* mutant cells were frequently non-flagellated and sporadically contained 1-2 flagella which were significantly shorter than those formed by the wild-type cells. This suggested serious disturbances in flagellum formation and/or function in the Rt2472 strain.

The type of cell flagellation is a strain-specific property, as shown by the fact that *R. leguminosarum* bv. *viciae* VF39 and *S. meliloti* 1021 strains were peritrichously flagellated similarly to Rt24.2, whereas *R. leguminosarum* bv. *viciae* 3841 had subpolar flagella. Also, the number of flagella might be strain-specific. For example, strain Rl3841 had 1–3 plain subpolar flagella while strain VF39 had 4–7 peritrichous flagella [28]. It had been established that mutations in certain flagellar genes significantly affected rhizobial motility [29, 58]. For instance, a mutation in *flaA* resulted in non-motile VF39 cells and extremely reduced the motility of Rl3841 cells, whereas mutations in *flaB* and *flaC* resulted in shorter flagellar filaments, which reduced swimming and swarming motility in the cells of both of these strains. However, mutations in other than motility-related genes could also affect this bacterial trait. For example, a *fabF* mutant of Rl3841, deficient in 27-hydroxyoctacosanoate-modified lipopolysaccharide, was impaired in motility, biofilm formation, and desiccation tolerance [59]. Motility of rhizobial cells is also affected by growth conditions such as agar concentration, inoculum size, temperature, and carbon source [29].

### Analysis of transcriptional fusions in the Rt2472 and Rt24.2 strains

**Fig. 5:**
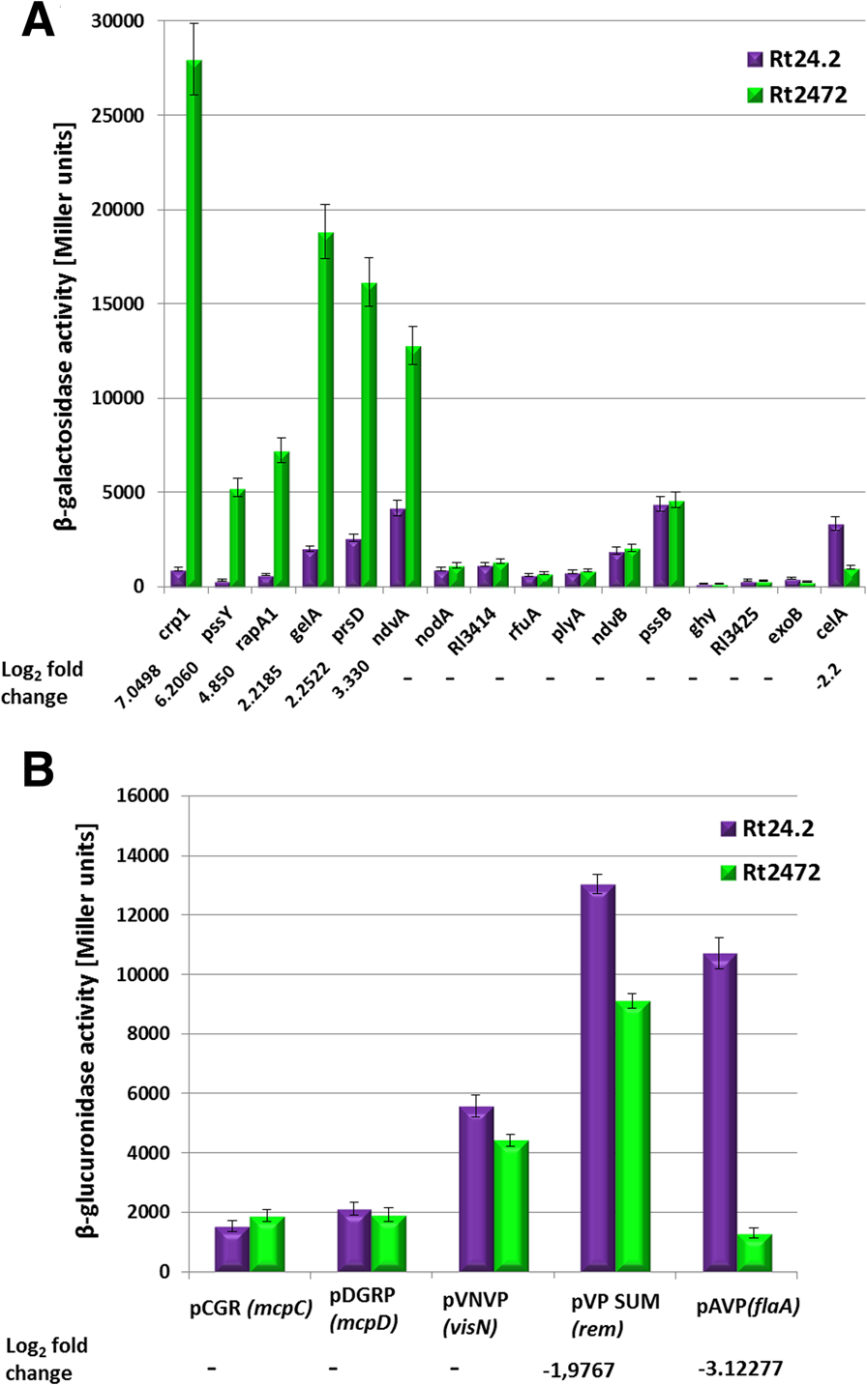
Transcriptional activity of rhizobial promoters in the *R. leguminosarum* bv. *trifolii* wild-type Rt24.2 and the *rosR* mutant Rt2472 strains. **a** β-galactosidase activity of the transcriptional fusions of representative genes encoding regulatory proteins, components of the transport systems, cell-surface components and enzymes for synthesis of polysaccharides; **b** β-glucuronidase activity of the transcriptional fusions of motility genes. The log_2_ fold changes for the individual genes obtained in the RNA-Seq analysis are given below the diagrams. Genes, for which differences in transcription levels between these backgrounds were not found, are marked with the symbol “-”

Several *R. leguminosarum* genes with different expression levels in the wild-type vs. the mutant background (*rapA1, prsD, pssY, crp1, celA, gelA, ndvA*), as well as those, whose expression was not affected by *rosR* (*plyA, rfuA, pssB, exoB, Rl3414, Rl3425, ghy, nodA, ndvB*), were chosen as representative genes for the experiment designed to validate the data obtained from transcriptomic analysis. The genes in these two groups showed a wide range of promoter strengths (from low to very high promoter activities). For this analysis, transcriptional fusions containing the promoter regions of these genes upstream of a promoterless *lacZ* were constructed (Additional file 8). In addition, plasmids with transcriptional fusions of motility-related genes (*mcpC, mcpD, flaA, visN* and *rem*), kindly provided by Prof C. Yost from the University of Regina (Canada) and Prof. M. Hynes from the University of Calgary (Canada), were included in the experiment. All these plasmid fusions were introduced into both Rt2472 and Rt24.2, and β-galactosidase or β-glucuronidase activity was measured (Fig. 5). Based on this analysis, it was found that promoters of the *ndvA, ndvB, prsD, pssB, celA, gelA, rem, visN, flaA, mcpD,* and *mcpC* genes were strong, since their activities in the wild-type strain were higher than 1500 Miller units. In contrast, a few of the studied genes (*pssY, exoB, crp1, rapA1, plyA, rfuA, nodA, Rl3414, Rl3425,* and *ghy*) possessed weak promoters (values below 1000 Miller units). When β-galactosidase activities for the individual fusions were compared between Rt2472 and Rt24.2, significant differences in the levels of transcription were found for *crp1, pssY, rapA1, gelA, prsD*, *ndvA, celA* (Fig. 5a), *flaA,* and *rem* genes (Fig. 5b). Among them, the highest Rt2472/Rt24.2 ratio was found for the following transcriptional fusions: *crp1-lacZ, pssY-lacZ, gelA-lacZ, rapA1-lacZ,* and *flaA-gusA*. On the other hand, similar levels of enzymatic activity in both of the strains tested were established for *rfuA, ndvB, plyA, pssB, nodA, Rl3425, Rl3414,* and *ghy,* indicating that RosR did not influence the expression of these genes.

In summary, the results obtained from the analysis of transcriptional fusions confirm that RosR is engaged in the regulation of the expression of *rapA1, prsD, flaA, crp1,* as well as *ndvA, gelA, celA,* and *pssY* involved in the synthesis of various rhizobial PS and other cell-surface components. These data are in conformity with those obtained in the RNA-Seq analysis. Previously, Bittinger and Handelsman, in their study of *R. etli* CE3, found that a mutation in a gene homologous to *R. leguminosarum* bv. *trifolii rosR* also affected the expression of several genes, including those involved in cellular metabolism (e.g. genes encoding coproporphyrinogen III oxidase, trehalose-phosphate synthase, and repressor of sucrose degradation operon) as well as those engaged in the synthesis and modification of polysaccharides (*exoY, prsD,* genes coding for cellulose synthase, CelR2 regulator of cellulose synthesis, and O-antigen acetylase) [60]. RosR of *R. etli*, similarly to *R. leguminosarum* RosR, functions as a negative regulator of gene expression, and this protein proved to be essential for nodulation competitiveness of *R. etli* on bean plants [45].

### Identification of RosR-box motifs in promoter regions of the genes differentially expressed in the *rosR* mutant and the wild-type strain

In order to establish which of the large group of genes differentially expressed in the mutant Rt2472 and the wild-type strain Rt24.2 were directly regulated by RosR, additional bioinformatics analyses were performed using a 22-nt-long RosR-box sequence and promoter regions of these genes. Sequence motifs showing a significant similarity to the RosR-box were found in the regulatory regions of many of these genes (195) (Additional file 8). Selected genes from this group and their RosR-box motifs are shown in Table 1. Among them, there were genes related to bacterial chemotaxis and motility (e.g. *Rt792_47, Rt792_70, Rt628_20* (*cheD*)*, Rt634_26* (*flbT*)*,* and *Rt628_11* (*fliN*)), transport and metabolism of carbon and nitrogen sources (e.g. *Rt620_74, Rt794_99, Rt617_43, Rt617_78, Rt619_162,* and *Rt794_91*), and synthesis of cell-surface components and envelope biogenesis (e.g. *Rt620_1* (*pssY*)*, Rt620_4, Rt780_45, Rt627_76* (*gelA*)*, Rt772_17* (*prsD*)). Moreover, several genes which encode proteins engaged in signal transduction and transcriptional regulators belonging to various families (Crp/Fnr, TetR, LuxR, LysR, AraC, and GntR) also possessed RosR-box motifs in their promoter regions. In this group, there were, among others, such genes as *Rt620_2* (*crp*)*, Rt648_25* (*lacI*)*, Rt634_21* (*rem*)*, Rt782_109* (*p-II*), *Rt646_16* (*raiI*)*, Rt648_50* (*phoB*) and *Rt628_39* (*phoR*)*,* which are involved in carbon, phosphate and nitrogen regulation, as well as motility and quorum sensing in *R. leguminosarum* [27, 28, 40, 49–51, 61]. These data indicate that RosR possibly controls a large group of these genes directly by binding to the sequence motifs located in their regulatory regions. However, in our study, there were many genes in which we did not identify motifs resembling the RosR-box; the expression of these genes may be indirectly regulated by RosR (probably mediated by the action of some transcriptional regulators encoded by COG K genes). Among the transcriptional regulators of *R. leguminosarum* mentioned above, the activator Rem, which together with LuxR-type regulators VisN and VisR positively controls the expression of many motility and chemotaxis genes, seems to be an interesting example of this relationship [49–51, 62]. Besides direct regulation of the transcription of several COG N genes, RosR was also found to indirectly affect the expression of some motility genes by modulating *rem* expression. Thus, RosR could be an important element of a second regulatory system controlling the expression of motility genes that are not under the control of the VisN/VisR-Rem regulatory cascade. The existence of such a system in *R. leguminosarum* was previously postulated by Tambalo and colleagues [61].

**Table 1:** Sequence motifs identified in regulatory regions of the genes differentially expressed in the *rosR* mutant and the wild-type strain, which show a significant similarity to the RosR-box sequence

Gene	Putative function of encoded protein product	COG	Log_2_ fold change	Sequence motif (5’-3’)^a^	Identity (%)	Distance to translation start site (nt)
*Rt617_78*	Amino acid ABC transporter substrate-binding protein	E	5.097	TGAAATCCATATCGACACTTCT	72.2	0
*Rt620_77*	Urea ABC transporter ATP-binding protein	E	3.502	CGGCTTCAAGGCGCTGAATTCG	66.7	61
*Rt619_162*	ABC-type dipeptide/oligopeptide transport system, permease	EP	−4.011	CAGAATCCGGCGGTGC-TTCCG	68.2	−153
*Rt619_164*	Peptide ABC transporter ATPase	EP	−4.438	CGCATTCTTCTCCTCTATATCG	66.7	−281
*Rt648_25*	LacI family transcriptional regulator	G	3.147	CGGCGGATAATCCTTGATCTCG	66.7	−176
*Rt794_16*	ABC transporter ATP-binding protein	G	2.968	GCGCTTCGTGGCGTGGATTTCG	66.7	56
*Rt794_99*	Sugar ABC transporter	G	2.053	CGGATTCCAGTAGGGATTTTCG	72.2	85
*Rt643_15*	Sugar ABC transporter substrate-binding protein	G	−3.744	CGGGGTCAGCAACTGCATTTCG	72.2	−83
*Rt620_14*	Glycerol 3-phosphate ABC transporter	G	2.133	CGAAATTTCTCTAAATATTTCC	66.7	−174
*Rt657_260*	Cell division protein FtsK	D	−3.211	CGGCGTCGAGATCTCGACATCG	66.7	−216
*Rt634_26*	Flagellar biosynthesis repressor FlbT	N	−3.247	CGCAAGGTCGCGCTGGAATTCC	66.7	74
*Rt634_27*	Flagellar basal body rod modification protein FlgD	N	−3.391	CAGAAGGCAACGCTGAATTACG	66.7	65
*Rt634_13*	Flagellin B	N	−4.601	TCCGATCTCGATTTAGCTTTCT	66.7	−170
*Rt634_5*	Flagellar basal body P-ring biosynthesis protein FlgA	N	−3.438	GGAAATCACCGAGCTGATCTCG	59.1	−99
*Rt628_11*	Flagellar motor switch protein FliN	NU	−4.579	CCGGCTGCGGGCATGGATTTCG	66.7	−13
*Rt761_94*	Methyl-accepting chemotaxis protein	NT	−3.966	CGCAAACGCAGTGTGTATTTGA	54.5	−124
*Rt628_20*	Chemotaxis protein CheD	NT	−2.353	CGAATTGGAAGATTGGCTGTCG	66.7	145
*Rt792_47*	Chemotaxis protein	NT	−2.587	CGGAATGCAGCTATCCATTCCC	66.7	−36
*Rt647_8*	Chemotaxis protein	NT	−3.651	TAAATTCTACGCCAAGATCGCC	66.7	127
*Rt628_24*	Chemotaxis protein CheW	NT	−3.050	CGGAGTCCAGCCCGGATTTCG	72.7	98
*Rt628_25*	Chemotaxis protein CheA	NT	−2.793	CGGAGTCCAGCCCCGGATTTCG	72.7	98
*Rt792_70*	Chemotaxis protein	NT	−3.025	CGGATGACATCAGAGGATTTCG	72.2	−298
*Rt628_22*	Chemotaxis protein CheB	NT	−3.030	CGGCGTCTATGAATGGGTGGAG	66.7	−40
*Rt620_4*	Glycosyl transferase	M	5.444	CGAATACCAAAGCCTGATTTCG	66.7	−76
*Rt620_5*	Polysaccharide biosynthesis protein	M	5.090	CTGGCGCGACTGCTCGATTTCG	66.7	59
*Rt794_35*	Polysaccharide biosynthesis protein	M	2.433	CGGCATCGGCCGGTAGATGACG	66.7	−191
*Rt768_57*	Peptidoglycan hydrolase	M	−2.657	CCGCATCGGCACGATGATTTCG	66.7	−243
*Rt679_3*	Glycosyl transferase	M	−3.806	CCGATTCACTATCTCGATCTCG	66.7	−137
*Rt621_6*	UDP-N-acetylmuramoyl-L-alanyl-D-glutamate synthetase	M	−2.028	CGAAATCCGCTGCGAGATATAA	66.7	130
*Rt623_92*	Glycosyl transferase	M	−2.450	TGAAGTCTTGGGACTGACATCA	66.7	−106
*Rt780_45*	Polysaccharide biosynthesis protein	M	7.890	TTGAAACGA-GCCTGGATTACG	63.6	291
*Rt627_76*	Polysaccharide biosynthesis protein GelA	M	2.218	CGGATCGAAGCG-TG-AATTCG	63.6	22
*Rt620_62*	Exopolysaccharide biosynthesis protein PssA	M	−1.170	CGAAATGCTGTTGTTTAGTTCC	54.5	11
*Rt772_12*	Glycosyl transferase PssC	M	−1.451	CTGAAGCTCCGGGGCGAAGCTCG	63.6	−125
*Rt780_150*	Signal peptide protein	H	3.925	ATGCATCAAATACTGCATGTCG	66.7	−239
*Rt622_32*	Ubiquinol oxidase subunit II	C	−6.942	CGAAAAATCCTTCTTGATTTCA	66.7	−231
*Rt620_122*	Iron transporter	P	−2.510	CGGAAGGCAGCGATGGTGTTCT	66.7	73
*Rt772_17*	PrsD component of type I secretion system	MU	2.252	CTGCAGCTACGGGTCCATCAAG	59.1	−50
*Rt646_16*	Acyl-homoserine-lactone synthase (RaiI)	T	6.049	AGCAATCTATGAATGGAATCGT	66.7	64
*Rt628_39*	Histidine kinase (PhoR)	T	3.583	GGTAATCTAGCACCGTAATTTG	66.7	−200
*Rt620_2*	Crp/Fnr family transcriptional regulator	T	7.049	TTTCAACTAAATGTAGACTTGA	66.7	−129
*Rt628_38*	Chemotaxis protein CheY	T	4.010	GAGGATCTCGCCGGGGATTTCC	66.7	−299
*Rt628_26*	Chemotaxis protein CheY	T	−3.129	CGGAACCAATGTCAGCATTGAG	66.7	81
*Rt782_109*	Nitrogen regulatory protein P-II	TE	3.152	CGGTATTCAGGGGCTGAC—-CG	63.6	80
*Rt648_50*	PhoB family transcriptional regulator	TK	3.900	GGTCGTCAAGGTGTTGAACCAG	50.0	−167
*Rt783_97*	Transcriptional regulator	K	4.442	CGGTGTCGGCCATCGGCTTTCG	66.7	−224
*Rt794_73*	TetR family transcriptional regulator	K	3.385	CGTCATCTCGATGACCATTTCG	66.7	49
*Rt764_77*	TetR family transcriptional regulator	K	2.774	TGAAATCTCCGCAGCCGTTCCA	66.7	40
*Rt780_87*	AraC family transcriptional regulator	K	2.340	GCAAATCAATCGATCGATCTCG	66.7	117
*Rt792_156*	LysR family transcriptional regulator	K	2.246	CAGGATCTGGAAGCCGATATCG	66.7	131
*Rt641_47*	LysR family transcriptional regulator	K	2.237	CGGAAGGTGTTTTCGGATGCCG	66.7	−267
*Rt636_12*	GntR family transcriptional regulator	K	2.044	CCGAAACTATCAGCGCGCTTCG	66.7	−45
*Rt764_77*	TetR family transcriptional regulator	K	2.774	TGAAATCTCCGCAGCCGTTCCA	66.7	40
*Rt634_21*	Transcriptional regulator	K	−1.776	TCAAATCGCTACCCAGATCTTA	66.7	−126
*Rt780_42*	Membrane protein	S	5.363	CGATATTTAATGATGGATACCA	66.7	−64
		RosR-box sequence		CGGAATCTAGGGGTGGATTTCG	-	-

^a^Identity of sequence motifs to the RosR-box was determined using 22-nt long sequences with the exception of their 4 nt in the middle of the sequence

Another possibility is that RosR regulates some genes of the RosR regulon, for which RosR-box motifs were not found in our study, by acting together with another regulatory protein, as evidenced for the RosR homolog in *S. meliloti,* MucR [58]. MucR single-handedly regulates the expression of many genes related to the synthesis of succinoglycan and the Nod factor, cell motility, and nitrogen fixation (e.g. *exoH, ExoX, exoYFQ, exoK, nodD*, and *fix* genes), and in a complex with WggR (ExpG) is involved in the regulation of galactoglucan synthesis genes [62–65].

In summary, data presented in this study suggest that RosR*,* similarly to *S. meliloti* MucR, plays an essential role in the regulatory network in *R. leguminosarum*, linking regulatory cascades associated with various environmental factors.

## Conclusions

In this study, we performed transcriptome profiling of the *rosR* mutant of *R. leguminosarum* bv. *trifolii* strain 24.2 using the next-generation RNA-Seq technology. This analysis allowed us to identify a large set of genes linked to motility, synthesis of cell-surface components, and several metabolic pathways, whose expression was affected by the *rosR* mutation. It was established that RosR is a global transcriptional regulator, which functions mainly as a repressor and is involved in several cellular processes. On the other hand, this protein is engaged in the positive regulation of many motility-related genes. The data presented in this study expand our knowledge of the role of RosR in the functioning of *R. leguminosarum* cells and provide insight into the regulatory network of this bacterium. Moreover, our study confirms the observation that the *rosR* mutation significantly affects the behavior of bacterial cells, as evidenced by their altered morphology, flagellation, motility, and aggregation ability.

## Methods

### Bacterial strains, plasmids, and culture conditions

The bacterial strains and plasmids used in this study are listed in Additional file 9. *R. leguminosarum* strains were grown in an energy-rich 79CA medium with 1 % glycerol as a carbon source and TY medium at 28 °C on a rotary shaker (160 rpm) as described previously [42]. *E. coli* strains were grown in Luria-Bertani (LB) medium at 37 °C [66]. When required, antibiotics were used at the following final concentrations: kanamycin, 40 μg ml^−1^; rifampicin, 40 μg ml^−1^; ampicillin, 100 μg ml^−1^; gentamicin, 10 μg ml^−1^; tetracycline, 10 or 5 μg ml^−1^; and nalidixic acid, 40 μg ml^−1^. The insertion of an antibiotic resistance cassette in the Rt2472 genome was stable even in the absence of kanamycin in the medium. Therefore, in order to exclude the influence of antibiotics on bacterial metabolism, they were only used for the growth and storage of the mutant strain on agar plates. Cultures of Rt2472, for a significant majority of the experiments (RNA isolation, motility and aggregation assays, determination of generation time, and flagella visualization) did not contain kanamycin. Tetracycline was used at a concentration of 10 μg ml^−1^ in two experiments (visualization of aggregation of the Rt24.2, Rt2472 and Rt2472(pBR24) strains carrying a pHC60 vector in fluorescent microscope, and β-galactosidase (β-glucuronidase) assays in the Rt24.2 and Rt2472 strains carrying transcriptional fusions). In the case of Rt2472(pRC24), the dose of tetracycline was reduced to a final concentration of 5 μg ml^−1^.

### DNA methods

Standard molecular techniques were used for DNA isolation, cloning, PCR reactions, ligation, transformation, and sequencing [66]. For PCR amplifications, the REDTaq Ready PCR Reaction Mix (Sigma, USA) was used. PCR products cloned into plasmid vectors were verified by sequencing using the BigDye terminator cycle sequencing kit (Applied Biosystems) and the ABI Prism 310 sequencer. Sequence analyses were done with the FASTA and BLAST programs from the National Center for Biotechnology Information (Bethesda, MD, USA) and the European Bioinformatic Institute (Hinxton, UK), respectively. Promoter prediction in upstream regions of the analyzed genes was done using BDGP Neural Network Promoter Prediction [67], whereas identification of promoter motifs was performed with the Malign program using *S. meliloti* CTTGAC-N_17-18_-CTATAT and *Escherichia coli* TTGACA-N_17-18_-TATAAT promoter consensuses [68, 69]. A RosR-box motif (5’-CGGAATCTAGGGGTGGATTTCG-3’), as a query, and the Rt24.2 genomic sequence were used to search for the genes regulated by RosR [68, 70]. Sequence motifs with a high similarity to the RosR-box sequence were identified using position weight matrices (PWM) and subsequently were annotated to the genes. The motifs located within a distance of 300-nt upstream to 150-nt downstream of the translation start site of the genes were selected. All calculations were done using the Biopython motif package and custom python scripts [71, 72].

### Total RNA isolation

For total RNA isolation, 25-ml cultures of Rt24.2 and Rt2472 were grown for 24 hours in 79CA medium in a rotary shaker. Then, the cultures were centrifuged for 10 min at 12,000 × *g*, and the bacterial pellet was suspended in 15 ml Trizol, shaken vigorously for 30 seconds, and incubated for 5 min at room temperature. After this period, 0.2 ml of chloroform was added per each 1 ml of Trizol used. The mixtures were shaken vigorously for 15 seconds, incubated for 8 minutes at room temperature, and subsequently centrifuged for 15 min at 12,000 × *g* at 4 °C. The RNA present in the water phase (12 ml) was transferred to a new tube and precipitated using 6 ml of isopropanol. Then, the mixtures were incubated at room temperature for 15 min and centrifuged for 15 min at 12,000 × *g* at 4 °C. The RNA pellets obtained were washed twice with 1 ml 75 % ethanol, mixed using a vortex mixer, centrifuged for 5 min at 7,500 × *g* at 4 °C, and dried in air for 10 min. Subsequently, the RNA pellets were dissolved in deionized RNase-free water and incubated for 10 min at 55 °C. The concentration of RNA isolated from the Rt24.2 and Rt2472 strains and their quality were determined using a NanoDrop 2000 spectrophotometer (Thermo Scientific, Wilmington, USA). Traces of DNA from the RNA solution were removed using a TURBO DNA-free Kit (Ambion, Life Technologies) according to the manufacturer’s instruction. Possible contamination of RNA by DNA was checked in PCR reactions using 2 primer pairs complementary to *pssA* (pssAG1f and pssA2r) and *pssY* (pssY5f and pssY5r) encoding glycosyl transferases in *R. leguminosarum* bv. *trifolii* as described earlier (Additional file 10) [73]. Then, rRNA from the total RNAs isolated from the Rt24.2 and Rt2472 strains was removed using a Ribo-Zero Magnetic Kit for Gram-Negative bacteria, containing magnetic beads (Epicentre, Illumina). The rRNA-depleted mRNA was precipitated using ice-cold ethanol at a ratio 3:1 (v/v, ethanol : RNA solution). The sample was gently mixed and incubated at −20 °C for an hour and then centrifuged for 30 minutes at 12,000 × *g*. The pellet was washed twice using ice-cold 70 % ethanol and centrifuged at 12,000 × *g* for 5 minutes. The mRNA pellet obtained was dissolved in deionized RNase- and DNase-free water, and the amount of RNA was quantified using a NanoDrop 2000 Spectrophotometer. RNA integrity was assessed using an Agilent Bioanalyzer 2100 and an RNA 6000 Pico Kit (Agilent Technologies).

### DNA sequencing and mapping, and analysis of RNA-Seq data

A draft genome of the Rt24.2 strain was sequenced using an Illumina MiSeq run (TruSeq DNA Technology) with paired-end reads. The initial preparation of the reads for analysis, including elimination of adapters and filtration of low quality reads, was performed using Cutadapt software [74]. *De novo* assembly of the Rt24.2 sequence was performed with the MaSurCA genome assembler using the default values for bacterial genomes [75]. The available switches were set for the assembly of bacterial genomes according to the software manual and the following parameters: ovlMerSize = 30, cgwErrorRate = 0.25, ovlMemory = 4GB, JF_SIZE = 100,000,000. As a result, the genomic sequence of the Rt24.2 strain was obtained in the form of 181 contigs with a total length of 7,653,217 bp (GC content = 60.6 %, average coverage 78.26×). In these sequences, coding regions were identified using the Prodigal program [76]. In total, 7,374 putative coding regions were found, for which amino acid sequences were obtained and, subsequently, matched to the highly homologous proteins available in databases using the Blastp program [70].

The mRNAs of Rt24.2 and Rt2472 were sequenced in 250-bp cycles using the MiSeq System with SBS technology (Illumina). Three independent biological experiments were performed for each of the two strains. Image analyses and basecalling were performed with the use of MiSeq Control Software (MCS 2.4.1.3) and an RTA component (RTA 1.18.54) (Illumina). The RNA-Seq data for Rt24.2 and Rt2472 were aligned with Bowtie2 [77] (within the Tophat package) using the reference Rt24.2 genome. Mapping parameters were set so that account could be taken of the strand-specific library option (fr-firststrand). The detection of novel splicing forms was disabled (option --no-novel-juncs). The median read number per CDS was above 1,000 (log value >3). For identification of genes with statistically significant differences in the expression levels between the Rt24.2 and Rt2472 strains, the Cufflinks\Cuffdiff software significance threshold value was set to α = 0.05 (using the Benjamini-Hochberg False Discovery Rate (FDR) correction) [78] (http://compbio.mit.edu/cummeRbund/manual_2_0.html). CDSs with FDR-corrected *p-values* for different expression between the *rosR* mutant and the wild-type lower than α = 0.05 were considered significant. As a result of the analysis, a list of genes differentially expressed in the tested strains with fold changes in the mutant strain versus the wild-type strain was obtained; the list also contained normalized expression FPKM (Fragments Per Kilobase of transcript per Million fragments mapped) values along with the Cuffdiff statistic test data.

To establish data quality and compare the expression levels between the Rt24.2 and Rt2472 strains, additional analyses were performed using cummeRbund (strain-specific repetition similarity calculated using a dendrogram with the Jensen-Shannon distance, distribution of FPKM values between the strains and between repetitions for an individual strain tested).

### Functional categorization of genes

The Clusters of Orthologous Groups database was used to classify genes differentially expressed in Rt2472 and Rt24.2 into functional categories (COG) [79].

### Construction of plasmids bearing transcriptional fusions with a reporter *lacZ* gene

In order to construct plasmids containing promoter regions of the analyzed genes cloned upstream of a promoterless *lacZ* gene, the broad-host-range vector pMP220 was used [80]. Using a genomic DNA of the Rt24.2 strain and 14 primer pairs (Additional file 10), PCR products encompassing the upstream regions of the *crp1, plyA, rfuA, pssB, ndvA, pssY, celA, gelA, rapA1, prsD, exoB, Rl3414, Rl3425,* and *ghy* genes were obtained. Subsequently, these amplicons were digested with appropriate restriction enzymes and cloned into the respective sites in the pUC19 (for the *plyA, rfuA, pssB, ndvA, pssY, celA, prsD, exoB, Rl3425,* and *ghy* promoters) or in the pMP220 plasmid (for the *crp1, gelA, rapA1,* and *Rl3414* promoters). The inserts of the latter plasmid group were re-cloned into the pUC19 vector. The nucleotide sequences of all these PCR products were verified by sequencing. Then, pUC19-derived fragments were re-cloned into the corresponding sites in pMP220, yielding plasmids which contained promoter fragments of the analyzed genes located upstream of the *lacZ* gene (Additional file 9). The resulting transcriptional fusions were introduced into *E. coli* S17-1 [81, 82] and subsequently into the Rt24.2 and Rt2472 strains by bi-parental conjugation. For this purpose, 24-h cultures of *E. coli* S17-1 derivatives carrying fusions (used as donor strains) and Rt24.2 and Rt2472 (used as recipient strains) were mixed at a 1:10 (v/v) ratio and centrifuged at 6,000 × *g* for 10 min. The bacterial pellet was suspended in 1 ml of sterilized water, and the mixture obtained was centrifuged for 10 min at 6,000 × *g*. Then, the pellet was suspended in 0.2 ml of sterilized water, and the bacterial mixture was placed on 79CA agar plates and incubated for 48 h at 28 °C. After this time, the bacteria were collected from the plates to 1 ml of sterilized water, and 0.1-ml aliquots were spread on 79CA agar plates supplemented with rifampicin and tetracycline. The transconjugants were obtained after one-week incubation at 28 °C.

### β-galactosidase and β-glucuronidase assays

The Rt24.2 and Rt2472 strains containing the transcriptional fusions were grown for 24 h in 79CA medium supplemented with tetracycline. The assays for β-galactosidase activity were carried out according to the protocol described by Miller [83] using 2-Nitrophenyl-β-D-galactopyranoside as a substrate (Sigma, USA). In the case of transcriptional fusions containing the reporter gene *gusA*, β-glucuronidase activity was determined using p-Nitrophenyl-β-D-glucuronide (Sigma, USA) [17]. The values reported are given in Miller units and are averages of at least three independent experiments.

### Motility assay

The ability of Rt24.2, Rt2472 and Rt2472(pRC24) cells to move was determined in 79CA medium with 0.3 % and 0.7 % agar. Plates containing 20 ml of these semi-solid or solid media were air-dried for 24 h and then inoculated with 5 μl of 24-h bacterial cultures in liquid 79CA medium of OD_600_ = 0.4. The plates were incubated at 25 °C for 3 and 21 days, respectively. Afterwards, the diameter of the injection site was measured. The experiment was repeated twice with three biological repetitions per each strain tested.

### Determination of generation time

In order to determine the generation time of Rt24.2, Rt2472 and Rt2472(pRC24) cells, 5 ml of liquid 79CA medium was inoculated with the bacteria to an optical density of OD_600_ = 0.15. Then, serial dilutions of the cultures were done after each 30 min during a 9-h period of growth. To this end, 100-μl aliquots of a particular dilution (from 10^−2^ to 10^−7^) were spread on 79CA agar plates. After three days of incubation at 28 °C, colonies were counted for each strain and each dilution tested. The number of bacterial cells present in the culture of an individual strain was calculated using the number of colonies × dilution factor × 10. The experiment was repeated twice with three biological repetitions per analyzed strain.

### Autoaggregation assay

The autoaggregation assay for the rhizobial strains was performed according to the method of Sorroche et al. [22] with a slight modification. Briefly, Rt24.2, Rt2472 and Rt2472(pRC24) were cultured in 5 ml of liquid 79CA medium on a rotary shaker for 24 h at 28 °C. The cultures were subsequently left to settle for 24 h without agitation. Afterwards, a 0.3-ml sample was taken from the upper part of the suspension, and its optical density (OD_600_) was measured (A_2_). Then, the cultures were vigorously mixed for 30 s, and OD_600_ was measured (A_1_). The autoaggregation percentage was calculated as follows: 100 * [1 − (A_2_/A_1_)]. The experiment was repeated twice with three biological repetitions per each strain tested.

### Transmission electron microscopy

Transmission electron microscopy was performed using a slightly modified procedure described by Tambalo et al. [29]. The Rt24.2, Rt2472 and Rt2472(pRC24) strains were grown on both solid 79CA and TY agar plates. The investigated strains (especially Rt24.2 and Rt2472(pRC24)) produced significantly smaller amounts of extracellular polysaccharides on TY than on 79CA medium. A formvar copper grid was gently placed on the plate containing bacteria and then incubated in 4 % ammonium molybdate for 1.5 min in order to dye the bacteria. The cells of each individual strain were observed using a LEO912AB transmission electron microscope.

## Additional files

Additional file 1:
**General features of the total sequenced and mapped reads for the** ***R. leguminosarum*** **bv.** ***trifolii*** **wild-type strain Rt24.2 and its derivative, the Rt2472** ***rosR*** **mutant* (A); (B) Dendrogram showing the similarity of the biological repetitions for Rt24.2 and Rt2472 measured using the Jensen–Shannon distance.** The samples for Rt24.2 are marked with wt_0, wt_1, and wt_2, whereas those for Rt2472 are marked with mut_0, mut_1, and mut_3; (**C**) Box plot of the distribution of FPKM values between three biological repetitions for the Rt24.2 and Rt2472 strains. (PDF 173 kb) Additional file 2:
**General features of FPKM values obtained for Rt24.2 and Rt2472 genes.** (**A**) Histogram of FPKM values for Rt24.2 and Rt2472 genes. The three biological repetitions for Rt24.2 are marked with wt_0, wt_1, and wt_2, whereas those for Rt2472 are marked with mut_0, mut_1, and mut_3; (**B**) Point diagram showing FPKM values of the individual genes obtained from Rt24.2 and Rt2472; (**C**) Point diagram showing log_2_ fold change values and *p-values* for the individual genes differentially expressed in Rt2472 and Rt24.2. (PDF 327 kb) Additional file 3:
**A list of coding sequences in** ***R. leguminosarum*** **bv.** ***trifolii,*** **for which differences in the expression levels between the Rt2472** ***rosR*** **mutant (mut) and the wild-type strain Rt24.2 (wt) were established (log**_**2**_ **fold change >2), with classification of the sequences into COG groups and putative functions of the encoded proteins.** (XLSX 148 kb) Additional file 4:
**List of putative CDS showing highest differences in the expression levels between the** ***rosR*** **mutant Rt2472 and the wild-type strain Rt24.2.** (DOCX 25 kb) Additional file 5:
**Autoaggregation of the** ***R. leguminosarum*** **bv.** ***trifolii*** **wild-type Rt24.2 strain, the** ***rosR*** **mutant Rt2472, and the Rt2472 strain after complementation by** ***rosR.*** (**A**) The autoaggregation ability of the analyzed strains determined after 24 h; (**B**) Cell morphology of the analyzed strains tagged with a *gusA* reporter gene (pHC60 plasmid) [84] in fluorescence microscopy (magnification, 200× and 630×). (PNG 547 kb) Additional file 6:
**Motility assay of** ***R. leguminosarum*** **bv.** ***trifolii*** **wild-type Rt24.2,** ***rosR*** **mutant Rt2472, and Rt2472(pRC24) cells on 0.3 % and 0.7 % 79CA agar plates in 3-day and 3-week experiments.** (DOCX 13 kb) Additional file 7:
**The cells of** ***R. leguminosarum*** **bv.** ***trifolii*** **wild-type Rt24.2, the** ***rosR*** **mutant Rt2472, and Rt2472(pRC24) visualized in transmission electron microscopy.** The strain Rt24.2 growing on 79CA (**A**) and TY medium (**B**); The strain Rt2472 growing on 79CA (**C**) and TY medium (**D**); The strain Rt2472(pRC24) growing on TY medium (**E** and **F**). Flagella are marked with red arrows and the extracellular layer surrounding bacterial cells is marked with a blue arrow. (PNG 1167 kb) Additional file 8:
**Sequence motifs identified in regulatory regions of the genes differentially expressed in the** ***rosR*** **mutant and the wild-type strain, which show a significant similarity to the RosR-box sequence.** (XLSX 26 kb) Additional file 9:
**Bacterial strains and plasmids used in this study.** (DOCX 16 kb) Additional file 10:
**Oligomeric primers used in this study.** (DOCX 14 kb)
