# Supplementary material for: Transcriptome profiling of a Rhizobium leguminosarum bv. trifolii rosR mutant reveals the role of the transcriptional regulator RosR in motility, synthesis of cell-surface components, and other cellular processes
Source: BMC Genomics. 2015 Dec 29;16:1111. doi: 10.1186/s12864-015-2332-4 (PMC4696191; doi:10.1186/s12864-015-2332-4)

A

Sample –strain	Total No. of raw reads	Total No. of reads mapped + Bowtie2	Average length	Total bases
Rt24.2-sample 1	7,136,686	6,517,669	164.57	1,072,627,304
Rt24.2-sample 2	5,397,418	4,649,312	166.37	773,538,582
Rt24.2-sample 3	5,649,550	4,939,791	155.34	767,380,752
Rt2472-sample 1	5,051,416	4,347,164	202.74	881,348,376
Rt2472-sample 2	4,666,754	4,231,022	186.15	787,611,441
Rt2472-sample 3	8,710,172	7,517,555	182.23	1,369,931,565

*The reads were mapped using Tophat (Bowtie2 aligner) with –no-novel-juncs option. For more details see the Methods section.

B

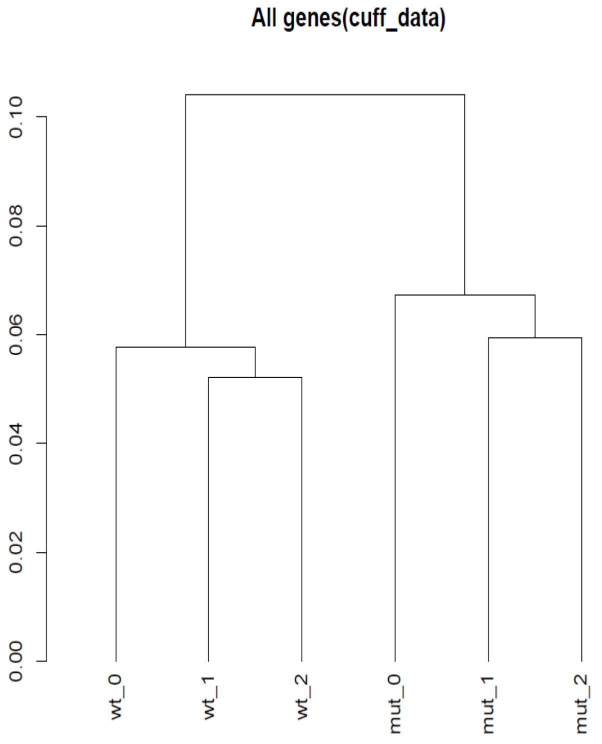

C

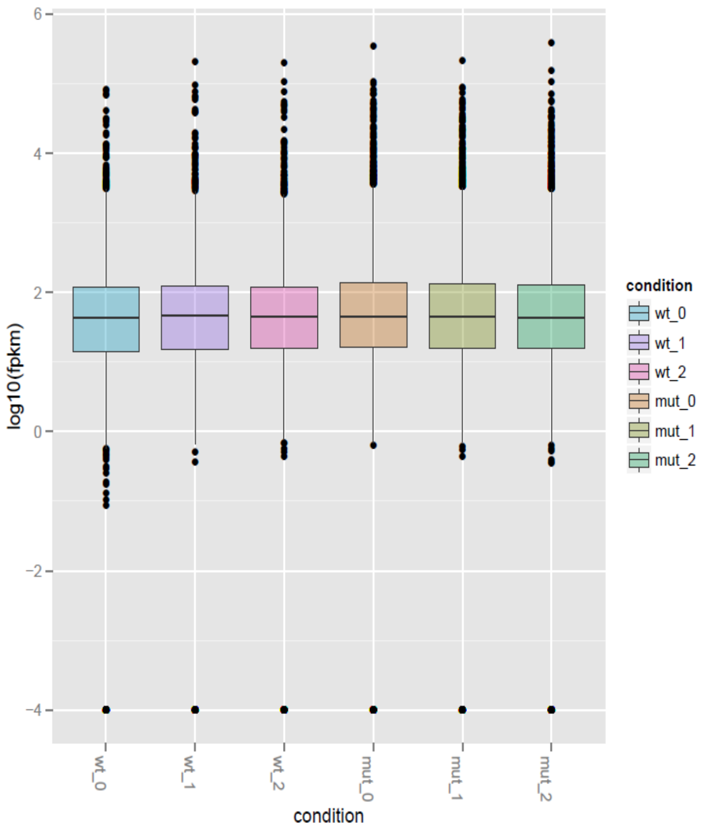

Supplement: Additional file 1: — General features of the total sequenced and mapped reads for the R. leguminosarum bv. trifolii wild-type strain Rt24.2 and its derivative, the Rt2472 rosR mutant* (A); (B) Dendrogram showing the similarity of the biological repetitions for Rt24.2 and Rt2472 measured using the Jensen–Shannon distance. The samples for Rt24.2 are marked with wt_0, wt_1, and wt_2, whereas those for Rt2472 are marked with mut_0, mut_1, and mut_3; (C) Box plot of the distribution of FPKM values between three biological repetitions for the Rt24.2 and Rt2472 strains. (PDF 173 kb) [file 12864_2015_2332_MOESM1_ESM.pdf]
