# Supplementary figures and images for: Transcriptome profiling of a Rhizobium leguminosarum bv. trifolii rosR mutant reveals the role of the transcriptional regulator RosR in motility, synthesis of cell-surface components, and other cellular processes
Source: BMC Genomics. 2015 Dec 29;16:1111. doi: 10.1186/s12864-015-2332-4 (PMC4696191; doi:10.1186/s12864-015-2332-4)

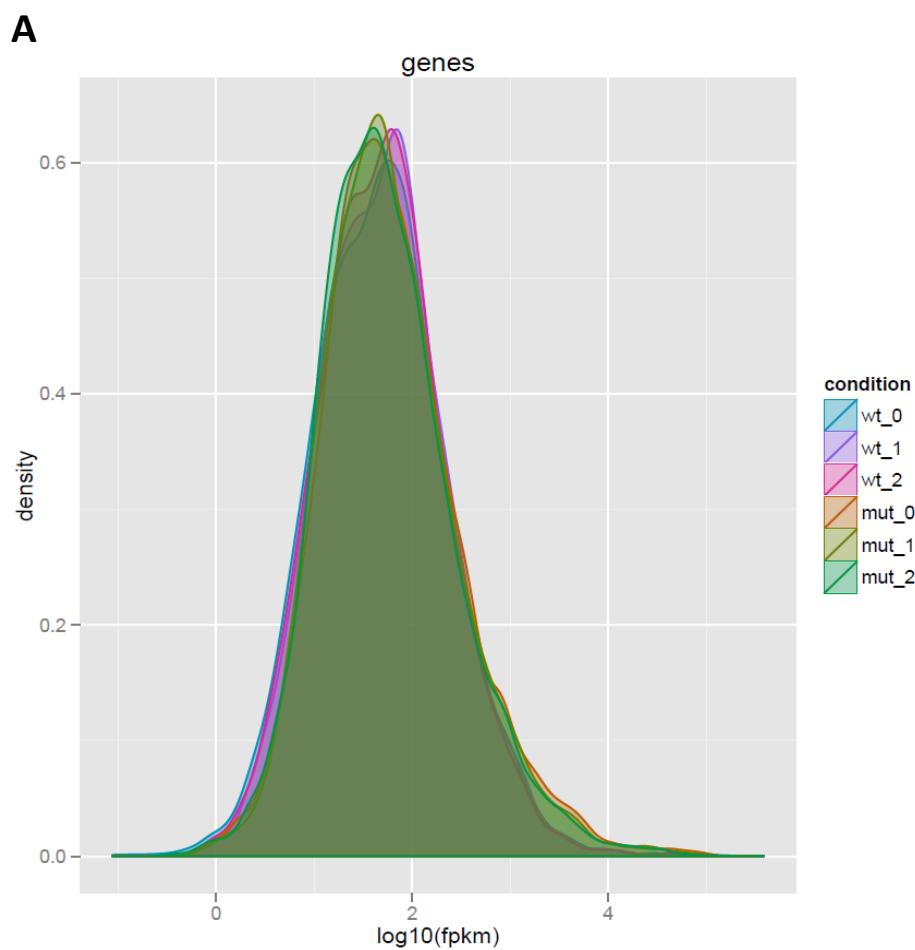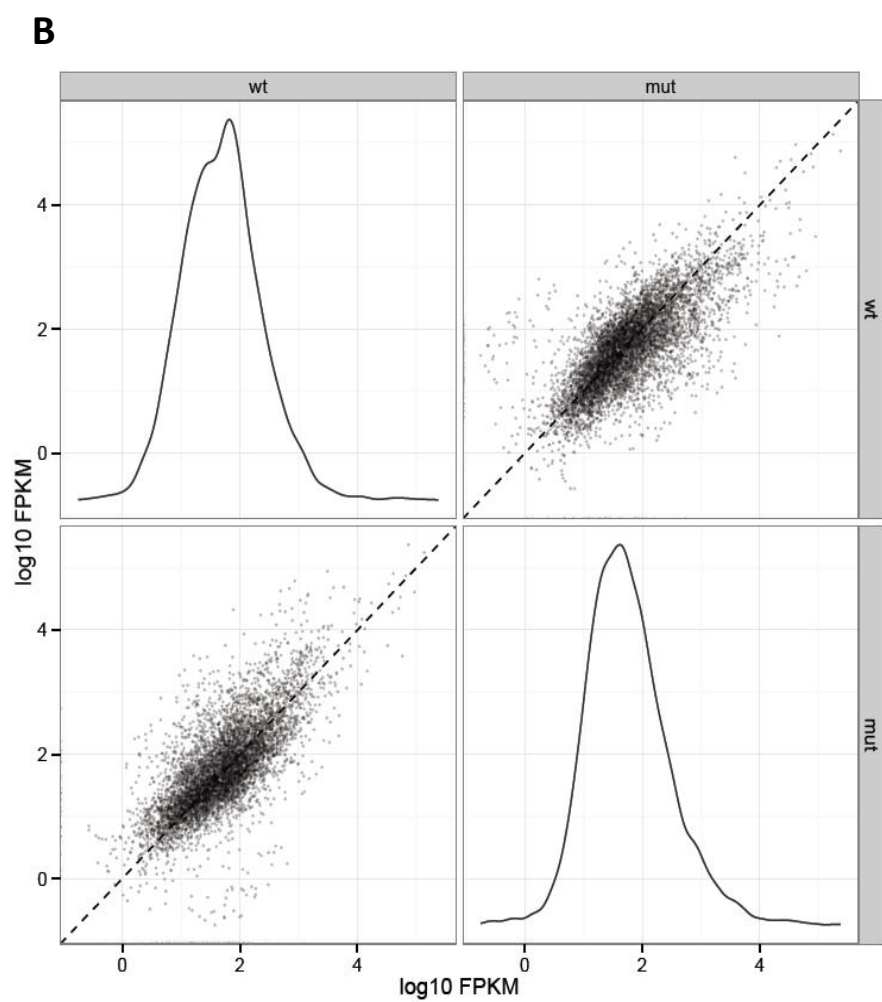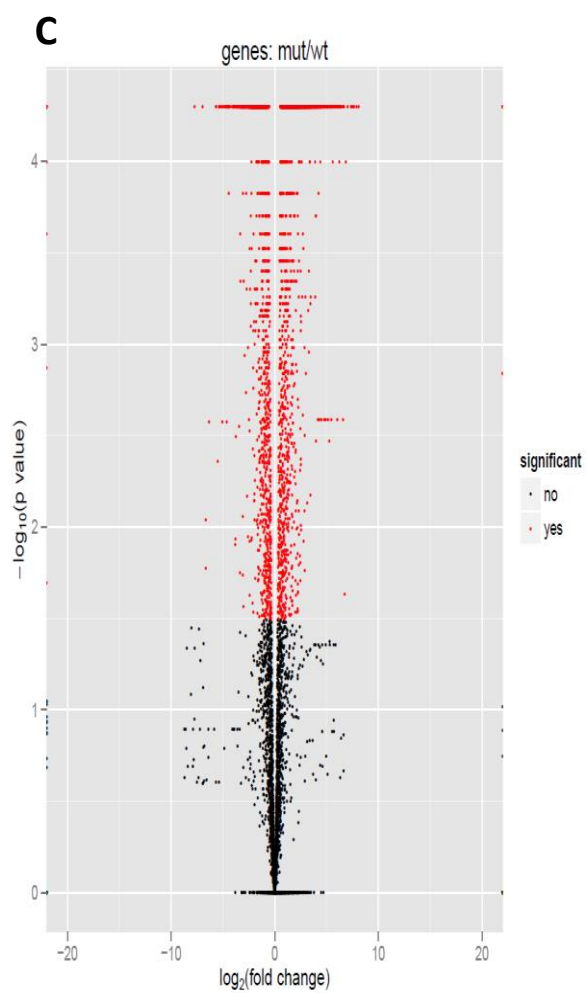

Supplement: Additional file 2: — General features of FPKM values obtained for Rt24.2 and Rt2472 genes. (A) Histogram of FPKM values for Rt24.2 and Rt2472 genes. The three biological repetitions for Rt24.2 are marked with wt_0, wt_1, and wt_2, whereas those for Rt2472 are marked with mut_0, mut_1, and mut_3; (B) Point diagram showing FPKM values of the individual genes obtained from Rt24.2 and Rt2472; (C) Point diagram showing log2 fold change values and p-values for the individual genes differentially expressed in Rt2472 and Rt24.2. (PDF 327 kb) [file 12864_2015_2332_MOESM2_ESM.pdf]

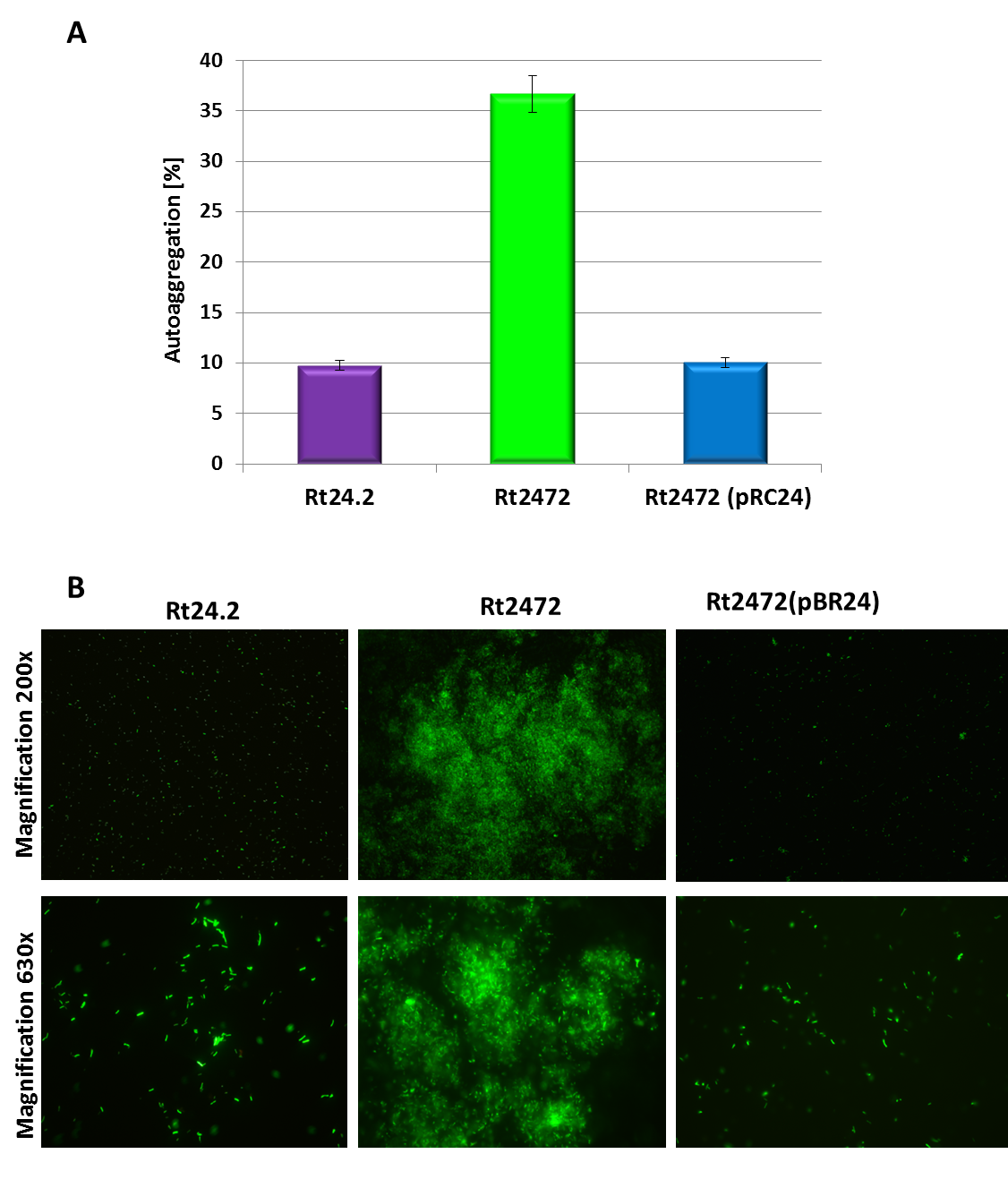

Supplement: Additional file 5: — Autoaggregation of the R. leguminosarum bv. trifolii wild-type Rt24.2 strain, the rosR mutant Rt2472, and the Rt2472 strain after complementation by rosR. (A) The autoaggregation ability of the analyzed strains determined after 24 h; (B) Cell morphology of the analyzed strains tagged with a gusA reporter gene (pHC60 plasmid) [84] in fluorescence microscopy (magnification, 200× and 630×). (PNG 547 kb) [file 12864_2015_2332_MOESM5_ESM.png]

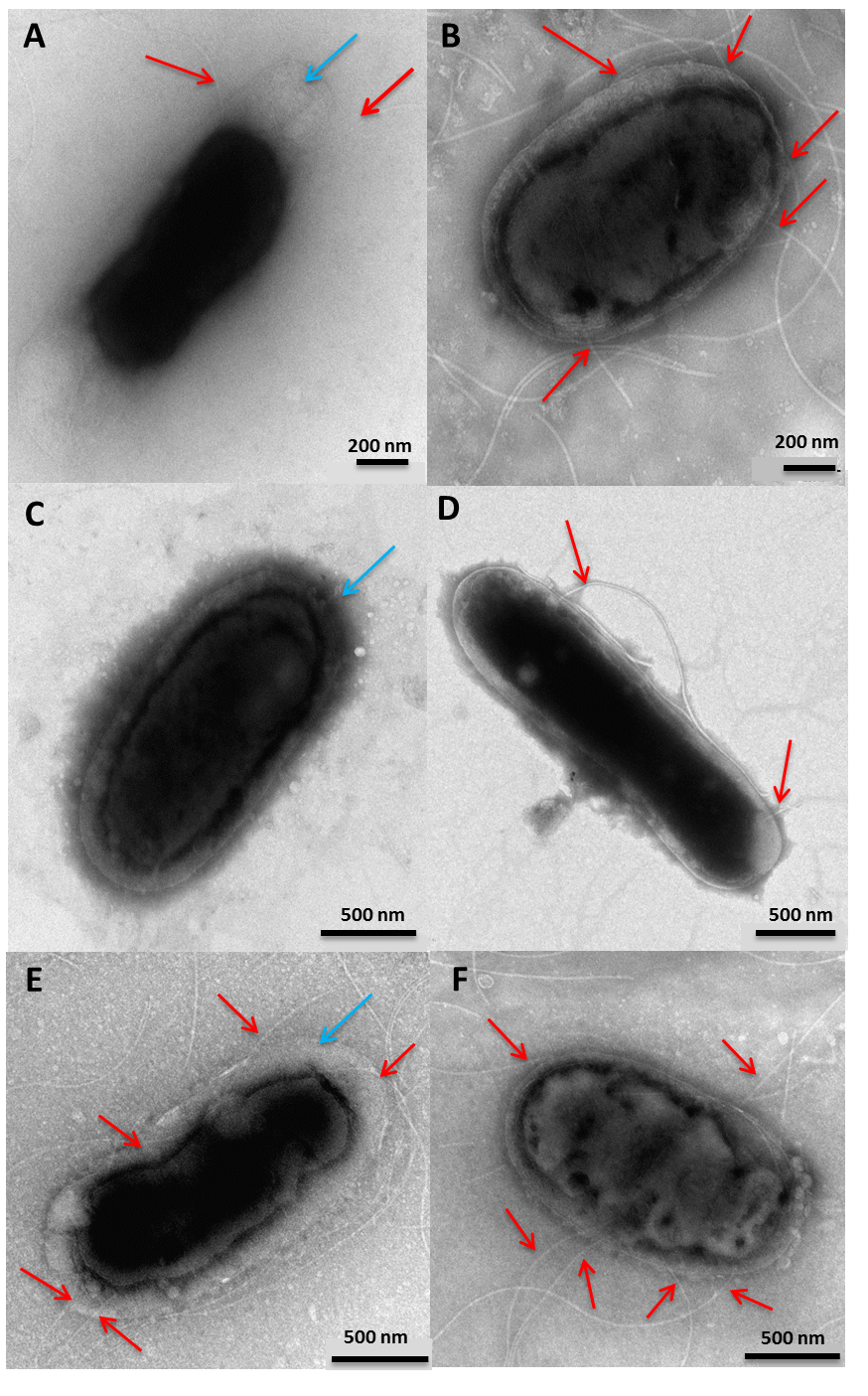

Supplement: Additional file 7: — The cells of R. leguminosarum bv. trifolii wild-type Rt24.2, the rosR mutant Rt2472, and Rt2472(pRC24) visualized in transmission electron microscopy. The strain Rt24.2 growing on 79CA (A) and TY medium (B); The strain Rt2472 growing on 79CA (C) and TY medium (D); The strain Rt2472(pRC24) growing on TY medium (E and F). Flagella are marked with red arrows and the extracellular layer surrounding bacterial cells is marked with a blue arrow. (PNG 1167 kb) [file 12864_2015_2332_MOESM7_ESM.png]
