# Supplementary material for: Transcriptome profiling of a Rhizobium leguminosarum bv. trifolii rosR mutant reveals the role of the transcriptional regulator RosR in motility, synthesis of cell-surface components, and other cellular processes
Source: BMC Genomics. 2015 Dec 29;16:1111. doi: 10.1186/s12864-015-2332-4 (PMC4696191; doi:10.1186/s12864-015-2332-4)
Supplement: Additional file 4: — List of putative CDS showing highest differences in the expression levels between the rosR mutant Rt2472 and the wild-type strain Rt24.2. (DOCX 25 kb) [file 12864_2015_2332_MOESM4_ESM.docx]

| **Additional file 4.** List of putative CDS showing highest differences in the expression levels between the *rosR* mutant Rt2472 and the wild-type strain Rt24.2. | | | | | | | | | | |  |  |  |  |
| --- | --- | --- | --- | --- | --- | --- | --- | --- | --- | --- | --- | --- | --- | --- |
| **Gene** | **COG name, CDS description, and putative function** | | | | | | | | | **log_2_ fold change** | | |  |  |
| **Energy production and conversion (C )** | | | | | | | | | | | | |  |  |
| *Rt622_33* | Heme/copper-type cytochrome/quinol oxidase, subunit 1 | | | | | | | | | -7.71 | | |  |  |
| *Rt622_32* | Heme/copper-type cytochrome/quinol oxidase, subunit 2 | | | | | | | | | -6.94 | | |  |  |
| *Rt794_89* | Glycerol-3-phosphate dehydrogenase | | | | | | | | | -4.78 | | |  |  |
| *Rt794_96* | Glycerol kinase | | | | | | | | | -4.68 | | |  |  |
| *Rt619_95* | Pyruvate/2-oxoglutarate/acetoin dehydrogenase complex, dehydrogenase (E1) component | | | | | | | | | -4.42 | | |  |  |
| *Rt793_18* | Tripartite-type tricarboxylate transporter, receptor component TctC | | | | | | | | | -4.30 | | |  |  |
| *Rt619_96* | Pyruvate/2-oxoglutarate dehydrogenase complex, dihydrolipoamide acyltransferase (E2) component | | | | | | | | | -3.99 | | |  |  |
| *Rt619_99* | Pyruvate/2-oxoglutarate dehydrogenase complex, dihydrolipoamide dehydrogenase (E3) component or related enzyme | | | | | | | | | -3.81 | | |  |  |
| *Rt642_40* | NADH:ubiquinone oxidoreductase subunit 3 (chain A) | | | | | | | | | 4.44 | | |  |  |
| *Rt629_73* | NAD(P)H-nitrite reductase, large subunit | | | | | | | | | 4.69 | | |  |  |
| *Rt642_41* | NADH:ubiquinone oxidoreductase 20 kD subunit (chain B) or related Fe-S oxidoreductase | | | | | | | | | 5.40 | | |  |  |
| *Rt615_6* | Lactate dehydrogenase or related 2-hydroxyacid dehydrogenase | | | | | | | | | 5.50 | | |  |  |
| *Rt615_5* | Phosphoenolpyruvate carboxylase | | | | | | | | | 7.37 | | |  |  |
| **Cell cycle control, cell division, chromosome partitioning (D)** | | | | | | | | | | | | |  |  |
| *Rt657_260* | DNA segregation ATPase FtsK/SpoIIIE proteins | | | | | | | | | -3.22 | | |  |  |
| *Rt626_125* | Cell division GTPase FtsZ | | | | | | | | | -3.12 | | |  |  |
| **Amino acid transport and metabolism (E )** | | | | | | | | | | | | |  |  |
| *Rt619_97* | Lysophospholipase L1 or related esterase | | | | | | | | | -4.46 | | |  |  |
| *Rt619_161* | ABC-type transport system, periplasmic component | | | | | | | | | -3.64 | | |  |  |
| *Rt663_8* | ABC-type transport system, periplasmic component | | | | | | | | | -3.54 | | |  |  |
| *Rt619_158* | Dihydrodipicolinate synthase/N-acetylneuraminate lyase | | | | | | | | | -4.79 | | |  |  |
| *Rt620_78* | ABC-type branched-chain amino acid transport system, ATPase component | | | | | | | | | 4.95 | | |  |  |
| *Rt617_78* | ABC-type branched-chain amino acid transport system, periplasmic component | | | | | | | | | 5.09 | | |  |  |
| *Rt793_309* | Uncharacterized protein involved in cysteine biosynthesis (CysZ-like protein) | | | | | | | | | 5.13 | | |  |  |
| *Rt646_12* | Glycine/D-amino acid oxidase | | | | | | | | | 6.25 | | |  |  |
| *Rt646_13* | Alanine racemase | | | | | | | | | 7.46 | | |  |  |
| **Amino acid transport and metabolism / Inorganic ion transport and metabolism (EP)** | | | | | | | | | | | | |  |  |
| *Rt619_165* | ABC-type dipeptide/oligopeptide/nickel transport system, ATPase component | | | | | | | | | -4.5 | | |  |  |
| *Rt619_164* | ABC-type dipeptide/oligopeptide/nickel transport system, ATPase component | | | | | | | | | -4.43 | | |  |  |
| *Rt619_163* | ABC-type dipeptide/oligopeptide/nickel transport system, permease component | | | | | | | | | -4.33 | | |  |  |
| *Rt619_162* | ABC-type dipeptide/oligopeptide/nickel transport system, permease component | | | | | | | | | -4.01 | | |  |  |
| **Inorganic ion transport and metabolism (P)** | | | | | | | | | | | | |  |  |
| *Rt626_8* | Periplasmic regulator RcnB of Ni and Co efflux | | | | | | | | | 5.04 | | |  |  |
| *Rt617_45* | ABC-type nitrate/sulfonate/bicarbonate transport system, permease component | | | | | | | | | 5.05 | | |  |  |
| *Rt617_43* | ABC-type nitrate/sulfonate/bicarbonate transport system, periplasmic component | | | | | | | | | 5.21 | | |  |  |
| *Rt629_74* | Nitrate/nitrite transporter NarK | | | | | | | | | 5.99 | | |  |  |
| *Rt617_44* | ABC-type nitrate/sulfonate/bicarbonate transport system, ATPase component | | | | | | | | | 6.59 | | |  |  |
| *Rt629_72* | Ferredoxin subunit of nitrite reductase | | | | | | | | | 5.15 | | |  |  |
| **Carbohydrate transport and metabolism (G)** | | | |  | | | | | |  | | |  |  |
| *Rt794_91* | ABC-type sugar transport system, ATPase component | | | | | | | | | -5.65 | | |  |  |
| *Rt794_90* | ABC-type sugar transport system, ATPase component | | | | | | | | | -5.63 | | |  |  |
| *Rt794_95* | ABC-type glycerol-3-phosphate transport system, periplasmic component | | | | | | | | | -5.37 | | |  |  |
| *Rt794_93* | ABC-type glycerol-3-phosphate transport system, permease component | | | | | | | | | -5.32 | | |  |  |
| *Rt794_92* | ABC-type sugar transport system, permease component | | | | | | | | | -5.21 | | |  |  |
| *Rt643_15* | ABC-type glycerol-3-phosphate transport system, periplasmic component | | | | | | | | | -3.74 | | |  |  |
| *Rt620_7* | ABC-type sugar transport system, ATPase component | | | | | | | | | 4.44 | | |  |  |
| *Rt620_6* | Beta-glucanase, GH16 family | | | | | | | | | 4.54 | | |  |  |
| *Rt794_14* | L-fucose mutarotase/ribose pyranase, RbsD/FucU family | | | | | | | | | 4.94 | | |  |  |
| *Rt615_7* | Fructosamine-3-kinase | | | | | | | | | 5.10 | | |  |  |
| *Rt620_29* | Arabinose efflux permease, MFS family | | | | | | | | | 5.55 | | |  |  |
| **Coenzyme transport and metabolism (H)** | | | | | |  | | | |  | | |  |  |
| *Rt646_10* | Adenosylmethionine-8-amino-7-oxononanoate aminotransferase | | | | | | | | | 4.55 | | |  |  |
| *Rt792_31* | Molybdopterin biosynthesis enzyme MoaB | | | | | | | | | 4.79 | | |  |  |
| *Rt615_63* | Dihydrofolate reductase, riboflavin biosynthesis protein RibD | | | | | | | | | 6.45 | | |  |  |
| **Lipid transport and metabolism (I)** | | | | | | |  | | |  | | |  |  |
| *Rt761_98* | Membrane-associated phospholipid phosphatase | | | | | | | | | 4.33 | | |  |  |
| *Rt628_94* | NAD(P)-dependent dehydrogenase, short-chain alcohol dehydrogenase family | | | | | | | | | 4.40 | | |  |  |
| **Transcription (K)** | | | | | | | | | | | | |  |  |
| *Rt763_92* | Transcriptional regulator, AcrR family | | | | | | | | | -6.63 | | |  |  |
| *Rt620_2* | Crp/Fnr family transcriptional regulator | | | | | | | | | 7.04 | | |  |  |
| *Rt793_310* | Transcriptional regulator, MurR/RpiR family | | | | | | | | | 4.36 | | |  |  |
| *Rt615_50* | Transcriptional regulator, MarR family | | | | | | | | | 4.38 | | |  |  |
| *Rt631_25* | Transcriptional regulator, AcrR family | | | | | | | | | 4.40 | | |  |  |
| *Rt780_151* | Transcriptional regulator, HxlR family | | | | | | | | | 4.44 | | |  |  |
| *Rt783_97* | RNA polymerase, sigma subunit (sigma70/sigma32) | | | | | | | | | 4.86 | | |  |  |
| *Rt620_47* | Transcriptional regulator, AcrR family | | | | | | | | | 5.09 | | |  |  |
| *Rt620_27* | Transcriptional regulator, MarR family | | | | | | | | | 5.28 | | |  |  |
| *Rt770_14* | Transcriptional regulator, AcrR family | | | | | | | | | 5.80 | | |  |  |
| *Rt617_23* | Transcriptional regulator, AraC family | | | | | | | | | 5.88 | | |  |  |
| *Rt646_9* | Transcriptional regulator, contains XRE-family HTH domain | | | | | | | | | 6.36 | | |  |  |
| *Rt643_8* | Response regulator, NarL/FixJ family | | | | | | | | | -5.47 | | |  |  |
| **Cell wall/membrane/envelope biogenesis (M)** | | | | | | | | | | | | |  |  |
| *Rt634_19* | Outer membrane protein assembly factor BamA | | | | | | | | | -4.75 | | |  |  |
| *Rt634_20* | Lytic murein transglycosylase, regulatory protein containing LysM/invasin domains | | | | | | | | | -4.41 | | |  |  |
| *Rt679_3* | Glycosyl transferase involved in cell wall biosynthesis | | | | | | | | | -3.80 | | |  |  |
| *Rt780_39* | Glycosyl transferase, GT2 family | | | | | | | | | 5.61 | | |  |  |
| *Rt640_62* | dTDP-4-amino-4,6-dideoxygalactose transaminase | | | | | | | | | 4.83 | | |  |  |
| *Rt620_5* | Membrane protein involved in the export of O-antigen and teichoic acid | | | | | | | | | 5.09 | | |  |  |
| *Rt620_4* | Glycosyl transferase involved in cell wall biosynthesis | | | | | | | | | 5.44 | | |  |  |
| *Rt794_34* | GDP-D-mannose dehydratase | | | | | | | | | 5.92 | | |  |  |
| *Rt620_3* | Glycosyl transferase involved in cell wall biosynthesis | | | | | | | | | 6.09 | | |  |  |
| *Rt620_1* | Sugar transferase involved in LPS biosynthesis (colanic, teichoic acid) | | | | | | | | | 6.20 | | |  |  |
| *Rt780_43* | Glycosyl transferase involved in cell wall biosynthesis | | | | | | | | | 6.61 | | |  |  |
| *Rt780_44* | Glycosyl transferase involved in cell wall biosynthesis | | | | | | | | | 7.64 | | |  |  |
| *Rt780_45* | Periplasmic protein involved in polysaccharide export | | | | | | | | | 7.89 | | |  |  |
| **Cell motility (N)** | | | | | | | | | | | | |  |  |
| *Rt634_14* | Flagellin C | | | | | | | | | -4.95 | | |  |  |
| *Rt634_13* | Flagellin B | | | | | | | | | -4.60 | | |  |  |
| *Rt628_11* | Flagellar motor switch/type III secretory pathway protein FliN | | | | | | | | | -4.57 | | |  |  |
| *Rt634_4* | Flagellar basal body rod protein FlgG | | | | | | | | | -4.45 | | |  |  |
| *Rt634_17* | Flagellar motor protein MotB | | | | | | | | | -4.02 | | |  |  |
| *Rt634_15* | Flagellin D | | | | | | | | | -3.86 | | |  |  |
| *Rt628_18* | Flagellar biosynthesis/type III secretory pathway, M-ring protein FliF/YscJ | | | | | | | | | -3.69 | | |  |  |
| *Rt634_6* | Flagellar basal body P-ring formation protein FlgI | | | | | | | | | -3.47 | | |  |  |
| *Rt634_5* | Flagella basal body P-ring formation protein FlgA | | | | | | | | | -3.43 | | |  |  |
| *Rt634_27* | Flagellar hook assembly protein FlgD | | | | | | | | | -3.39 | | |  |  |
| *Rt634_26* | Flagellar biosynthesis regulator FlbT | | | | | | | | | -3.24 | | |  |  |
| *Rt634_8* | Flagellar basal body L-ring formation protein FlgH | | | | | | | | | -3.22 | | |  |  |
| *Rt634_12* | Flagellin A | | | | | | | | | -3.12 | | |  |  |
| *Rt634_23* | Flagellar hook-associated protein FlgK | | | | | | | | | -3.12 | | |  |  |
| *Rt644_53* | Flp pilus assembly protein, protease CpaA | | | | | | | | | 6.04 | | |  |  |
| **Signal transduction mechanisms (T)** | | | | | | | | | | | | |  |  |
| *Rt622_21* | Adenylate cyclase, class 3 | | | | | | | | | -6.32 | | |  |  |
| *Rt628_27* | Anti-anti-sigma regulatory factor (antagonist of anti-sigma factor) - chemotaxis protein CheX | | | | | | | | | -3.42 | | |  |  |
| *Rt628_26* | CheY chemotaxis protein or a CheY-like REC (receiver) domain | | | | | | | | | -3.12 | | |  |  |
| *Rt628_21* | Chemotaxis protein CheY | | | | | | | | | -3.10 | | |  |  |
| *Rt628_22* | Chemotaxis response regulator CheB | | | | | | | | | -3.03 | | |  |  |
| *Rt794_46* | DNA-binding response regulator, NarL/FixJ family, contains REC and HTH domains | | | | | | | | | 5.38 | | |  |  |
| *Rt783_116* | Serine/threonine protein phosphatase PrpC | | | | | | | | | 4.45 | | |  |  |
| *Rt647_18* | Ca2+-binding protein, EF-hand superfamily | | | | | | | | | 5.50 | | |  |  |
| *Rt630_30* | Nucleotide-binding universal stress protein, UspA family | | | | | | | | | 5.90 | | |  |  |
| *Rt646_16* | N-acyl-L-homoserine lactone synthase | | | | | | | | | 6.04 | | |  |  |
| **Signal transduction mechanisms \ Cell motility (TN)** | | | | | | | |  | |  | | |  |  |
| *Rt761_94* | Methyl-accepting chemotaxis protein | | | | | | | | | -3.96 | | |  |  |
| *Rt647_8* | Methyl-accepting chemotaxis protein | | | | | | | | | -3.65 | | |  |  |
| *Rt784_108* | Methyl-accepting chemotaxis protein | | | | | | | | | -3.29 | | |  |  |
| **Secondary metabolites biosynthesis, transport and catabolism (Q)** | | | | | | | | | | | | |  |  |
| *Rt763_93* | O-Methyltransferase involved in polyketide biosynthesis | | | | | | | | | -4.62 | | |  |  |
| *Rt618_102* | Lysine/ornithine N-monooxygenase | | | | | | | | | -3.68 | | |  |  |
| *Rt618_103* | Non-ribosomal peptide synthase, component F | | | | | | | | | -3.60 | | |  |  |
| **General function prediction only (R )** | | | | | | | | | | | | |  |  |
| *Rt643_19* | 4,5,-dihydroxyphthalate dehydrogenase | | | | | | | | | -6.62 | | |  |  |
| *Rt622_38* | NAD(P)/FAD-binding protein YdhS | | | | | | | | | -5.08 | | |  |  |
| *Rt762_187* | Peptide synthase | | | | | | | | | 4.32 | | |  |  |
| *Rt784_136* | TolB amino-terminal domain (function unknown) | | | | | | | | | 4.40 | | |  |  |
| *Rt622_94* | Protein containing caspase domain - serine/threonine protein kinase | | | | | | | | | 4.41 | | |  |  |
| *Rt624_109* | Short-chain dehydrogenase | | | | | | | | | 4.88 | | |  |  |
| *Rt780_135* | Membrane protein YeaQ/YmgE, transglycosylase-associated protein family | | | | | | | | | 5.05 | | |  |  |
| **Function unknown (S)** | | | | | | | | | | | | |  |  |
| *Rt794_94* | Small integral membrane protein | | | | | | | | | -3.77 | | |  |  |
| *Rt780_14* | Major facilitator transporter | | | | | | | | | -3.64 | | |  |  |
| *Rt784_62* | Major facilitator transporter | | | | | | | | | 4.32 | | |  |  |
| *Rt619_134* | Conserved protein YjbJ, UPF0337 family | | | | | | | | | 4.36 | | |  |  |
| *Rt673_20* | Conserved peptide-binding protein YraI | | | | | | | | | 4.39 | | |  |  |
| *Rt615_49* | Conserved aldehyde-activating protein | | | | | | | | | 4.44 | | |  |  |
| *Rt645_42* | Membrane protein | | | | | | | | | 4.71 | | |  |  |
| *Rt624_104* | Membrane protein | | | | | | | | | 4.77 | | |  |  |
| *Rt622_42* | Membrane protein 4.83 | | | | | | | | | | | | | 4.83103 |
| *Rt780_42* | Membrane protein | | | | | | | | | 5.36 | | |  |  |
| *Rt620_138* | Membrane protein | | | | | | | | | 5.76 | | |  |  |
| *Rt780_40* | Membrane protein | | | | | | | | | 6.76 | | |  |  |
| *Rt632_44* | Signal peptide protein | | | | | | | | | 6.87 | | |  |  |
| **Defense mechanisms (V)** | | | | | | | | | | | | |  |  |
| *Rt615_24* | Multidrug resistance efflux pump | | | | | | | | | 4.52 | | |  |  |
| *Rt620_28* | Multidrug resistance efflux pump | | | | | | | | | 4.68 | | |  |  |
| *Rt780_58* | FMN-containing flavoprotein, pyridoxamine 5'-phosphate oxidase superfamily | | | | | | | | | 5.19 | | |  |  |
| **Intracellular trafficking, secretion, and vesicular transport (U)** | | | | | | | | |  |  | | |  |  |
| *Rt772_19* | Autoaggregation protein | | | | | | | | | 4.85 | | |  |  |
| *Rt784_63* | Signal peptide protein | | | | | | | | | 5.39 | | |  |  |
| **Mobilome: prophages, transposons (X)** | | | | |  | | | | |  | | |  |  |
| *Rt615_25* | | Transposase IS3 | | | | | | | | 5.47 | | |  |  |
| **Hypothetical proteins** | | |  | | | | | | |  | | |  |  |
| *Rt761_93* | Hypothetical protein | | | | | | | | | -4.49 | | |  |  |
| *Rt785_24* | Hypothetical protein | | | | | | | | | -3.93 | | |  |  |
| *Rt768_1* | Hypothetical protein | | | | | | | | | -3.80 | | |  |  |
| *Rt617_62* | Hypothetical protein | | | | | | | | | 4.32 | | |  |  |
| *Rt629_20* | Hypothetical protein | | | | | | | | | 4.49 | | |  |  |
| *Rt646_17* | Hypothetical protein | | | | | | | | | 4.51 | | |  |  |
| *Rt617_63* | Hypothetical protein | | | | | | | | | 4.61 | | |  |  |
| *Rt780_55* | Hypothetical protein | | | | | | | | | 4.63 | | |  |  |
| *Rt780_54* | Hypothetical protein | | | | | | | | | 4.63 | | |  |  |
| *Rt780_53* | Hypothetical protein | | | | | | | | | 4.79 | | |  |  |
| *Rt692_1* | Hypothetical protein | | | | | | | | | 4.98 | | |  |  |
| *Rt780_41* | Hypothetical protein | | | | | | | | | 5.08 | | |  |  |
| *Rt631_31* | Hypothetical protein | | | | | | | | | 5.61 | | |  |  |
| *Rt656_4* | Hypothetical protein | | | | | | | | | 5.69 | | |  |  |
| *Rt656_5* | Hypothetical protein | | | | | | | | | 6.06 | | |  |  |
| *Rt786_50* | Hypothetical protein | | | | | | | | | 6.15 | | |  |  |
| *Rt621_92* | Hypothetical protein | | | | | | | | | 6.43 | | |  |  |
| *Rt619_103* | Hypothetical protein | | | | | | | | | 6.69 | | |  |  |
| *Rt623_59* | Hypothetical protein | | | | | | | | | 8.10 | | |  |  |
