# Supplementary material for: Transcriptome profiling of a Rhizobium leguminosarum bv. trifolii rosR mutant reveals the role of the transcriptional regulator RosR in motility, synthesis of cell-surface components, and other cellular processes
Source: BMC Genomics. 2015 Dec 29;16:1111. doi: 10.1186/s12864-015-2332-4 (PMC4696191; doi:10.1186/s12864-015-2332-4)
Supplement: Additional file 6: — Motility assay of R. leguminosarum bv. trifolii wild-type Rt24.2, rosR mutant Rt2472, and Rt2472(pRC24) cells on 0.3 % and 0.7 % 79CA agar plates in 3-day and 3-week experiments. (DOCX 13 kb) [file 12864_2015_2332_MOESM6_ESM.docx]

**Additional file 6.** Motility assay of *R. leguminosarum* bv. *trifolii* wild-type Rt24.2, *rosR* mutant Rt2472, and Rt2472(pRC24) cells on 0.3% and 0.7% 79CA agar plates in 3-day and 3-week experiments.

| **Strain** | **Agar concentration (%)** | **Migration zone diameter (mm)** | |
| --- | --- | --- | --- |
|  |  | **3 days** | **3 weeks** |
| **Rt24.2 (wild-type)** | 0.3 | 24 ± 0.94 | 70 ± 4.11 |
|  | 0.7 | 13 ± 1.25 | 37 ± 1.08 |
| **Rt2472(*rosR^-^)*** | 0.3 | 12 ± 0.82 | 26 ± 1.22 |
|  | 0.7 | 10 ± 0.82 | 18 ± 1.63 |
| **Rt2472(pRC24)** | 0.3 | 20 ± 2.23 | 78 ± 2.1 |
|  | 0.7 | 13 ± 2.38 | 39 ± 4.5 |
