# Supplementary material for: Transcriptome profiling of a Rhizobium leguminosarum bv. trifolii rosR mutant reveals the role of the transcriptional regulator RosR in motility, synthesis of cell-surface components, and other cellular processes
Source: BMC Genomics. 2015 Dec 29;16:1111. doi: 10.1186/s12864-015-2332-4 (PMC4696191; doi:10.1186/s12864-015-2332-4)
Supplement: Additional file 9: — Bacterial strains and plasmids used in this study. (DOCX 16 kb) [file 12864_2015_2332_MOESM9_ESM.docx]

**Additional file 9.** Bacterial strains and plasmids used in this study

| **Strains and plasmids** | | | **Relevant characteristics^*^** | | **Reference** | | | | |
| --- | --- | --- | --- | --- | --- | --- | --- | --- | --- |
| *R. leguminosarum* bv. *trifolii* | | | |  |  | | | | |
| Rt24.2 | Wild type, Rif^r^, Nx^r^ | | | | | [42] | | | |
| Rt2472 | Rt24.2 with a mutation in the *rosR* gene, Rif^r^, Km^r^ | | | | | [42] | | | |
| *E. coli* |  | | | | |  | | | |
| DH5α | *supE*44 Δ*lac*U169 (φ80 *lacZ*Δ M15) *hsdR*17 *recA*1*endA*1*gyrA*96 *thi*-1 *relA*1 | | | | | [66] | | | |
| S17-1 | 294, *thi*, RP4-2-Tc::Mu-Km::Tn*7* | | | | | [84] | | | |
| **Plasmids** |  | | | | |  | | | |
| pUC19 | Cloning and sequencing vector, Ap^r^ | | | | | [66] | | | |
| pMP220 | IncP, *mob*, promoterless *lacZ*, Tc^r^ | | | | | [81] | | | |
| pFUS1P | pFUS1 with *par* cassette, promoterless *gusA*, Tc^r^ | | | | | [26] | | | |
| pHC60 | RK2 with a reporter gene *gfp*, Tc^r^ | | | | | [83] | | | |
| pRC24 | pRK7813 with 1,174-bp *Bam*HI fragment containing *rosR* of Rt24.2, Tc^r^ | | | | | | | | [40] |
| pBR24 | pBBR1MCS-5 with 1,174-bp *Bam*HI fragment containing *rosR* of  Rt24.2, Gm^r^ | | | | | | | | [40] |
| pUC-C1 | pUC19 carrying the 0.84-kb *Bam*HI-*Pst*I fragment of the *crp1* promoter | | | | | | | This work | |
| pUC-P2 | pUC19 carrying the 0.85-kb *EcoR*I-*Pst*I fragment of the *plyA* promoter | | | | | | | This work | |
| pUC-R3 | pUC19 carrying the 0.5-kb *EcoR*I-*Pst*I fragment of the *rfuA* promoter | | | | | | | This work | |
| pUC-B4 | pUC19 carrying the 0.6-kb *EcoR*I-*Pst*I fragment of the *pssB* promoter | | | | | | | This work | |
| pUC-N5 | pUC19 carrying the 0.3-kb *EcoR*I-*Pst*I fragment of the *ndvA* promoter | | | | | | | This work | |
| pUC-Y6 | pUC19 carrying the 0.76-kb *EcoR*I-*Pst*I fragment of the *pssY* promoter | | | | | | | This work | |
| pUC-3414 | pUC19 carrying the 0.84-kb *Bam*HI-*Pst*I fragment of the promoter region of a UDP-phosphate galactose phosphotransferase gene | | | | | | | This work | |
| pUC-3425 | pUC19 carrying the 0.48-kb *EcoR*I-*Pst*I fragment of the promoter region of an ABC transporter ATP-binding protein gene | | | | | | | This work | |
| pUC-CE9 | pUC19 carrying the 0.72-kb *EcoR*I-*Pst*I fragment of the *celA* promoter | | | | | | This work | | |
| pUC-G10 | pUC19 with the 0.8-kb *BamH*I-*Xba*I fragment of the *gelA* promoter | | | | | | This work | | |
| pUC-R11 | pUC19 with the 0.9-kb *BamH*I-*Xba*I fragment of the *rapA1* promoter | | | | | | This work | | |
| pUC-PR12 | | pUC19 with the 0.85-kb *EcoR*I-*Xba*I fragment of the *prsD* promoter | | | | | This work | | |
| pUC-E13 | pUC19 with the 0.62-kb *EcoR*I-*Xba*I fragment of the *exoB* promoter | | | | | | This work | | |
| pUC-EG14 | | pUC19 with the 0.62-kb *EcoR*I-*Pst*I fragment of the *ghy* promoter for a glycosyl hydrolase family 5 | | | | | This work | | |
| pCRP1 | pMP220 with the 0.84-kb *Bgl*II-*Pst*I fragment of the *crp1* promoter | | | | | This work | | | |
| pPLY2 | pMP220 with the 0.85-kb *EcoR*I-*Pst*I fragment of the *plyA* promoter | | | | | This work | | | |
| pRFU3 | pMP220 with the 0.5-kb *EcoR*I-*Pst*I fragment of the *rfuA* promoter | | | | | This work | | | |
| pPSS4 | pMP220 with the 0.6-kb *EcoR*I-*Pst*I fragment of the *pssB* promoter | | | | | This work | | | |
| pNDV5 | pMP220 with the 0.3-kb *EcoR*I-*Pst*I fragment of the *ndvA* promoter | | | | | This work | | | |
| pSY6 | pMP220 with the 0.76-kb *EcoR*I-*Pst*I fragment of the *pssY* promoter | | | | | This work | | | |
| pM3414 | pMP220 with the 0.84-kb *Bgl*II-*Pst*I fragment of UDP-phosphate galactose phosphotransferase gene *Rl3414* | | | | | This work | | | |
| pM3425 | pMP220 carrying the 0.48-kb *EcoR*I-*Pst*I fragment of the ABC transporter ATP-binding protein gene *Rl3425* | | | | | This work | | | |
| pCEL9 | pMP220 with the 0.72-kb *EcoR*I-*Pst*I fragment of the *celA* promoter | | | | | This work | | | |
| pGEL10 | pUC19 with the 0.8-kb  *Bgl*II-*Xba*I fragment of the *gelA* promoter | | | | | This work | | | |
| pRAP11 | pMP220 with the 0.9-kb *Bgl*II-*Xba*I fragment of the *rapA1* promoter | | | | | This work | | | |
| pPRS12 | pMP220 with the 0.85-kb *EcoR*I-*Xba*I fragment of the *prsD* promoter | | | | | | This work | | |
| pEXO13 | pMP220 with the 0.62-kb *EcoR*I-*Xba*I fragment of the *exoB* promoter | | | | | | This work | | |
| pEGL14 | pMP220 with the 0.62-kb *EcoR*I-*Pst*I fragment of the *ghy* promoter | | | | | This work | | | |
| pMPA221 | pMP220 with the 125-bp *EcoR*I-*Pst*I fragment of the *nodA* promoter | | | | | [82] | | | |
| pNdvB | pFUS1P carrying the *ndvB-gusA* transcriptional fusion | | | | | [59] | | | |
| pDGRP | pFUS1P carrying the *mcpD-gusA* transcriptional fusion | | | | | [26] | | | |
| pAVP | pFUS1P carrying the *flaA-gusA* transcriptional fusion | | | | | [61] | | | |
| pVNVP | pFUS1P carrying the *visN-gusA* transcriptional fusion | | | | | | [61] | | |
| pSVP SUM | | pFUS1P carrying the *rem-gusA* transcriptional fusion | | | | | [61] | | |
| pCGR | pRK7813 carrying the *mcpC-gusA* transcriptional fusion | | | | | | [26] | | |

Nx^r^, nalidixic acid resistance, Rif^r^, rifampicin resistance, Tc^r^, tetracycline resistance, Ap^r^, ampicillin resistance, Km^r^, kanamycin resistance, Gm^r^, gentamicin resistance.
