# Supplementary material for: Transcriptome profiling of a Rhizobium leguminosarum bv. trifolii rosR mutant reveals the role of the transcriptional regulator RosR in motility, synthesis of cell-surface components, and other cellular processes
Source: BMC Genomics. 2015 Dec 29;16:1111. doi: 10.1186/s12864-015-2332-4 (PMC4696191; doi:10.1186/s12864-015-2332-4)
Supplement: Additional file 10: — Oligomeric primers used in this study. (DOCX 14 kb) [file 12864_2015_2332_MOESM10_ESM.docx]

**Additional file 10.** Oligomeric primers used in this study

| **Gene** | **Primer name** | | | **Nucleotide sequence (5′-3′)*** | | **Tm (°C)** | |
| --- | --- | --- | --- | --- | --- | --- | --- |
| ***crp1*** | | CRP-5 | GGTCTCCAAGATCTTCCGATTAAGAA | | 56.4 | |  |
| ***crp1*** | | CRP-6 | GATGAATTCCCTGCAGTCGGGCA | | 58.8 | |  |
| ***plyA*** | | PlyA-76 | TCCCGGCTCTAGACTCAAGGATTCGACA | | 62.9 | |  |
| ***plyA*** | | PlyA-77 | CTAAGTGAATTCTGACCTTTAGCGAGCTG | | 60.1 | |  |
| ***rfuA*** | | RfuA-17 | GAAGATATCTGCAGGAGAGGCGTC | | 59.1 | |  |
| ***rfuA*** | | RfuA-18 | CAACACGATGAATTCCGGCAGGAC | | 59.1 | |  |
| ***pssB*** | | PssB-19 | GATGATACCTGCAGCCTCCTCGTC | | 60.8 | |  |
| ***pssB*** | | PssB-20 | TGGTTCTTGAATTCTCCGTTTCAGAGAAC | | 58.7 | |  |
| ***ndvA*** | | NdvA-23 | TGTCCTCGCTGCAGCGGGTAATGT | | 60.8 | |  |
| ***ndvA*** | | NdvA-24 | CGGCGACGAATTCCCAGCAATCAG | | 60.8 | |  |
| ***pssY*** | | PssY-60 | ACGATCAACTGCAGCGGGGAAAG | | 58.8 | |  |
| ***pssY*** | | PssY-90 | CAGGAAAGGTACCCGTCGTCGATG | | 60.8 | |  |
| ***celA*** | | Cel-80 | AGGCACGGAATTCACCTCGACGGTATC | | 62.8 | |  |
| ***celA*** | | Cel-81 | TCTGACCCTGCAGGGAGGATGAGGTAA | | 62.8 | |  |
| ***gelA*** | | Gel-85 | TCGCAAAGAATTCCTTCGGCATCTATAT | | 57.0 | |  |
| ***gelA*** | | Gel-86 | AGCGATTTCTAGAAAAAGCGGCGCCA | | 59.5 | |  |
| ***rapA1*** | | RapA-78 | CGCCTTGAATTCCCAAAGATACTGGAA | | 58.2 | |  |
| ***rapA1*** | | RapA-79 | GCAGATTTCTAGAAATGGCGTCGGTTT | | 58.2 | |  |
| ***prsD*** | | Prs-9 | ACCGGGGAATTCGGCGTCATCCA | | 60.6 | |  |
| ***prsD*** | | Prs-72 | TGAAACGTCTAGAGCATCAACGCAAGTA | | 58.5 | |  |
| ***exoB*** | | ExoB-7 | TATGGCGCTTCCGAATTCAAGACCG | | 59.3 | |  |
| ***exoB*** | | ExoB-88 | GACGATGTTCTAGAGAGTGTTCGA | | 55.7 | |  |
| ***Rl3414*** | | RL-61 | CTGGTGCAGATCTCCCCGTCCATA | | 60.8 | |  |
| ***Rl3414*** | | RL-62 | ACCAATTGCTGCAGCTTGTCGAGG | | 59.1 | |  |
| ***Rl3425*** | | RL-67 | CAAGGATCAGAATTCTCTTTCTCCCCCA | | 59.9 | |  |
| ***Rl3425*** | | RL-68 | TTCGGCCGCTGCAGCACGAGATC | | 62.4 | |  |
| ***ghy*** | | Egl-55 | AGCGCCGGCTGCAGCCGCTCCCA | | 67.8 | |  |
| ***ghy*** | | Egl-56 | GCCGACGCTGAATTCAACGGCGCG | | 64.2 | |  |
| ***pssA*** | | pssAG1f | CGCACATGCGAAAGATTTGCTGCG | | 59.1 | |  |
| ***pssA*** | | pssA2r | CCAGATCGAGGAATTCCCGACGTA | | 59.1 | |  |
| ***pssY*** | | pssY5f | GTCGTCGATGACGATGCGGCTGTT | | 60.8 | |  |
| ***pssY*** | | pssY5r | GAAACTATGTGCTTCCCATGTCATCG | | 58.0 | |  |

*The sequences for the *Eco*RI, *Xba*I, *Sac*I, *Sph*I, *Kpn*I, *Bgl*II, *Bam*HI and *Pst*I restriction sites are underlined.
